# ETPP: An Efficient Traversal Path Planning Framework for Autonomous Robot Exploration in Unknown Environments

**DOI:** 10.3390/s26196129

**Published:** 2026-09-27

**Authors:** Lingli Yu, Chengxiang Peng, Jiawei Luo, Kaijun Zhou, Zhixiang Chen

**Affiliations:** 1 School of Automation, Central South University, No. 932 Lushan South Road, Changsha 410083, China; llyu@csu.edu.cn (L.Y.); jiaweiluo@csu.edu.cn (J.L.); 234611104@csu.edu.cn (Z.C.); 2 School of Intelligent Engineering and Intelligent Manufacturing, Hunan University of Technology and Business, No. 569 Yuelu Avenue, Changsha 410205, China; zkj@hutb.edu.cn

**Keywords:** autonomous exploration, road map, traversal path planning, mobile robot, 68T40, 90C27, 90C35

## Abstract

Autonomous robot exploration in unknown environments is often constrained by the inefficiency of locally optimal strategies that evaluate frontier candidates individually for next-best-view (NBV) selection. To address this limitation, we propose an Efficient Traversal Path Planning (ETPP) framework. During exploration, frontiers are dynamically generated, and a roadmap representing the environmental backbone topology is incrementally constructed. Subsequently, a traversal path planning module is introduced, which employs an adaptive iterative cyclic crossover COOT algorithm to determine the global traversal path across frontiers. This global path is then combined with a local exploration path planned via an information entropy-based algorithm. For NBV selection, we design a distance–orientation evaluation function. Finally, robot navigation within the unknown environment is achieved using an Angle-based Pedestrian Grid Soft Actor–Critic (APG-SAC) motion controller. Mutual information is utilized for online NBV updates to minimize exploration cost. Simulation results demonstrate that ETPP reduces path length and time consumption by 15.95% and 21.04%, respectively, compared to state-of-the-art methods. Real-world experiments further validate the practical feasibility of the proposed framework.

## 1. Introduction

Autonomous exploration refers to the ability of robots to collect and model unknown environmental information using sensor data and software algorithms. It holds the potential to replace humans in dangerous environments [1], including environmental monitoring [2,3], deep-sea exploration [4], and dangerous rescue [5]. Moreover, it can free humans from simple and repetitive work, such as frequent manual mapping in highly variable and dynamic environments, which are time-consuming and require the operator’s technical knowledge. However, the limitation of robot battery capacity and the lack of prior knowledge of the environment bring great challenges to autonomous exploration [6]. Thus, it is imperative to study efficient methods for autonomous exploration.

During autonomous exploration, decision-making and planning are two crucial modules that guide the direction of exploration and generate collision-free trajectories for the robot to complete the exploration [7]. Various methods exist for determining next best view (NBV), including calculating the Euclidean distance [8] between the current position of the robot and the frontier or directly evaluating the information gain offered by the frontier [9]. The former tends to construct the map at a lower path cost quickly. In contrast, the latter first constructs the main parts of the environment and then supplements the details.

The studies above select NBV based on a single criterion. Some studies propose comprehensive evaluations considering path cost and information gain [10]. However, these methods are short-sighted, and their performance in large-scale scenes is often unsatisfactory. The larger environment size results in higher computational complexity for path planning. Furthermore, frequent path replanning is unavoidable due to the uncertainty of the environment.

This paper proposes an Efficient Traversal Path Planning (ETPP) framework for autonomous robot exploration. Within this framework, a hybrid frontier generation strategy rapidly identifies frontiers by leveraging polar coordinate characteristics extracted from laser point cloud data. Recognizing that frontiers inherently embody unexplored regions, the core challenge of autonomous exploration is reformulated as the problem of optimally visiting all frontiers. This transforms autonomous exploration into a frontier traversal planning problem, specifically modelled as a Frontier Traveling Salesman Problem (TSP). While the TSP has been employed for coverage path planning in multi-robot search missions [11], existing approaches typically operate under the assumption of static environments with predefined waypoints. The extension of TSP-based planning to online frontier traversal in partially explored environments remains largely unaddressed, which motivates our ACXCOOT-based traversal planning framework.

To enable efficient traversal planning, we introduce a potential field-based incremental method for roadmap construction, dynamically building a topological representation of the environment’s backbone during exploration. Global paths between frontiers are planned on this roadmap, with path costs quantified using Euclidean distance. We then solve the Frontier TSP problem using the Adaptive Iterative Cyclic Crossover COOT (ACXCOOT) algorithm to obtain the optimal frontier visitation sequence.

For next-best-view (NBV) selection, we design a distance–orientation evaluation function. Subsequently, the robot is navigated towards the selected NBV using an Angle-based Pedestrian Grid Soft Actor–Critic (APG-SAC) motion controller. Furthermore, an NBV updating mechanism, utilizing mutual information to reassess frontier utility during traversal, is implemented to minimize exploration path costs. The main contributions of this paper are summarized as follows:

- A novel traversal path planning method that integrates global path optimization via ACXCOOT for frontier traversal with local exploration planning based on information entropy. This hierarchical approach achieves computational efficiency while maintaining long-range planning foresight.
- An APG-SAC-based motion control method incorporating an Angle-based Pedestrian Grid (APG) module to emulate human-like observation patterns, enabling smooth, collision-free, and stable robot navigation.
- An online potential field-based roadmap construction technique that synchronously builds a sparse topological map representing environmental backbone connectivity during exploration, facilitating rapid path cost evaluation between frontiers.
- Comprehensive experimental validation demonstrating the qualitative and quantitative advantages of ETPP through extensive simulations and real-world testing.

## 2. Related Work

### 2.1. Frontier-Based Theory

The autonomous exploration method based on the frontier theory can be traced back to the work [8], which defines frontiers as discrete points at the intersection of known and unknown areas. The robot continuously explores the environment while moving towards the frontier, detecting and generating frontiers. This process repeats until the entire environment is explored. However, the frontier detection algorithm based on breadth-first search in [8] exhibits high time complexity. In contrast, the wavefront frontier detection algorithm [12] based on graph search detects frontiers solely in the known area, greatly reducing the time complexity. Similar to most graph search-based algorithms, the efficiency of the wavefront frontier detection algorithm sharply decreases in large-scale environments. On the other hand, the fast frontier detection algorithm [12] demonstrates higher computational efficiency as it solely processes real-time data from sensors. Subsequently, several algorithms for frontier detection have been proposed. Umari et al. [13] propose an autonomous exploration method based on multi rapidly exploring random tree (RRT), where RRT is used not for path planning but for finding frontiers. The execution of RRT can take place in parallel with robot motion, and the design of multiple RRT strategies further accelerates the frontier search process.

Once frontiers are generated, evaluating the costs and benefits associated with the robot moving toward each frontier becomes essential. The most common method for frontier evaluation involves considering either the path cost [14], information gain [9], or a combination of both. The importance of path cost and information gain can be adjusted using weighting coefficients after normalization to eliminate the influence of dimensions [15]. Additionally, the information gain calculation can be enhanced by heuristics biased sampling guided by the semantic information of the environment, which re-evaluates the importance of different areas based on recognized objects [16]. However, whether the constructed map is available depends on the accuracy of the localization. Fang et al. [17] introduce an indicator of localization accuracy into the evaluation function, whereby localization accuracy is measured by the percentage of the obstacle areas detected by the sensor. The aforementioned frontier evaluation strategies adopt a myopic approach, selecting a single NBV at each decision step based on local cost–benefit analysis. While computationally efficient, such greedy decision-making often leads to suboptimal global trajectories, particularly in large-scale environments where robots may revisit explored regions or miss efficient multi-frontier routes [18]. This limitation has motivated a paradigm shift from single-step frontier selection to global traversal planning, where the objective is to determine an optimal visitation order for all detected frontiers. Kim and Kim [11] formulate coverage path planning as a TSP for multi-robot cooperative search, demonstrating that global optimization of visitation order significantly reduces mission completion time compared to greedy strategies. However, their approach assumes static environments with predefined coverage waypoints, making it inapplicable to unknown environments where frontier locations are revealed incrementally. He et al. [19] address multi-robot navigation through hierarchical motion planning with graph attention neural networks, yet their method relies on known environment maps and does not consider the online nature of frontier detection during exploration. The integration of efficient global traversal optimization with real-time unknown environment exploration—accounting for dynamically revealed frontiers and incrementally constructed roadmaps—remains an open challenge that this paper seeks to address.

### 2.2. Information-Based Theory

Autonomous exploration based on information theory constructs an optimization problem by calculating the mutual information between the current map and the expected map and then solving for the path that maximizes information gain [20]. Efficient computation of mutual information is essential due to the numerical integration required to calculate the mutual information of the target area. In this case, Cauchy–Schwarz quadratic mutual information [21] and fast Shannon mutual information [22] provide computationally efficient closed-form solutions. Henderson et al. [23] further propose a faster computation method based on the recursive expression of Shannon mutual information. However, these methods may decrease accuracy due to their estimation approach. To address this, Xu et al. [24] propose an information calculation method based on the continuous belief distribution on the grid map to strike a balance between solution speed and accuracy.

### 2.3. Learning-Based Theory

Although autonomous exploration based on information theory is theoretically complete, the optimization problems constructed by such methods pose challenges in finding the optimal solution. With the emergence of deep reinforcement learning (DRL) to address the curse of dimensionality in Q-learning algorithms [25], DRL-based autonomous exploration methods have gradually attracted researchers’ attention. Bai et al. [26] construct a supervised learning problem to reduce the Shannon entropy of the map. Niroui et al. [27] decompose the autonomous exploration process into three modules: detection, decision-making, and planning. The detection module generates frontiers, which are then input into the decision-making module composed of neural networks to determine NBV. Subsequently, the NBV is sent to the planning module. In contrast to the studies mentioned above, Li et al. [28] propose merging the detection and decision-making modules, reducing the reliance on manual rules and enhancing the robot’s ability to handle complex scenarios. Compared with combining traditional methods with DRL, learning-based exploration policies that integrate mapping, global goal selection, and local control in a modular and hierarchical manner achieve higher exploration efficiency, since the learned components are only responsible for the subproblems that are difficult to formulate analytically, while analytical planners handle the well-posed geometric parts [29].

## 3. System Overview

The proposed framework of ETPP, as shown in Figure 1, consists of the external inputs, frontier generation and road map construction module (Section 4), traversal path planning module (Section 5), and path tracking and updating module (Section 6). The frontier generation and road map construction module generate frontiers and incrementally construct the road map based on external input data. Then, the traversal path planning module solves TSP for the frontiers and selects the NBV. Finally, the APG-SAC motion controller guides the robot towards the NBV while executing the path and NBV update functions. The exploration is considered complete only when there are no more frontiers.

## 4. Frontier Generation and Road Map Construction

Unlike previous work [8], this paper introduces a hybrid frontier generation strategy designed based on the polar coordinate characteristics of point cloud data acquired by laser. This method enables faster frontier detection and provides more comprehensive information. Additionally, adopting an incremental road map construction approach reduces the cost of traversal path planning (Section 5).

### 4.1. Hybrid Strategy Frontier Generation

Laser point cloud data consists of detection distance and detection azimuth, denoted as $P_{s}=\left\{\left(d_{i},\varphi_{i}\right)|i\in 0,\ldots ,n_{s}\right\}$, where $n_{s}=\left(\varphi_{\max}-\varphi_{\min}\right)/res_{s}$ and $\varphi \in \left[\varphi_{\min},\varphi_{\max}\right]$ represent the detection range of the laser. For a continuous stream of $n_{f1}$ point cloud, if it  satisfies

(1) $$d_{i}=d_{m},\;i\in \left\{j,\ldots ,j+n_{f1}\right\},\;j\in \left\{0,\ldots ,n_{s}-n_{f1}\right\},$$

it is considered that there are no obstacles in the sector region formed by these $n_{f1}$ points and the current position $p_{\mathrm{bot}}$ of the robot. Therefore, a frontier $p_{f1}\left(d_{f1},\varphi_{f1}\right)$ can be generated, as shown by the orange point in Figure 2, where $d_{f1}=d_{m}-c_{1}$ and $\varphi_{f1}=\left(\varphi_{j+n_{f1}}+\varphi_{j}\right)/2$, $c_{1}$ is a constant used to maintain a safe distance between $p_{f1}$ and the obstacles, as shown in Figure 2. The green point is the position determined by the original $d_{m}$, which collides with the obstacle represented by the undetected dashed line. After setting the safety distance $c_{1}$, the green point turns into an orange point, which ensures the safety of the robot.

However, relying solely on $p_{f1}$ for autonomous exploration is insufficient. It is also necessary to generate frontier $p_{f2}\left(d_{f2},\varphi_{f2}\right)$ based on the variation in detection distance $∥d_{i+1}-d_{i}∥\geq 2\times r_{\mathrm{bot}}$, as shown by the blue point in Figure 2, where $d_{f2}=\left(d_{i}+d_{i+1}\right)/2$, $\varphi_{f2}=\varphi_{i}$, and $r_{\mathrm{bot}}$ denotes the robot’s radius. Obviously, the information gain of $p_{f1}$ is higher, so when $p_{f1}$ exists, $p_{f2}$ is not considered. Otherwise, the information gain of $p_{f2}$ is evaluated for filtering, and the remaining $p_{f2}$ is utilized for subsequent steps.

### 4.2. Road Map Construction Based on Potential Field

The construction of the road map G(V,E) is performed incrementally to ensure real-time maintenance efficiency. Initially, nodes V(x,y) are generated through uniform point scattering, with a constant distance of $c_{V}$ between neighboring pairs of nodes. Then, the nodes overlapping with obstacles are eliminated, and the remaining nodes are optimized using a potential field function U(V):

(2) $$U\left(V\right)=U_{\mathrm{tar}}\left(V\right)+U_{\mathrm{obs}}\left(V\right)=0+e^{-D_{\mathrm{obs}}\left(V,p_{\mathrm{obs}}\right)},$$

where $U_{\mathrm{tar}}\left(V\right)$ represents the attractive field of the target, which is 0 in this case, and $U_{\mathrm{obs}}\left(V\right)$ represents the repulsive field of obstacles, with $D_{\mathrm{obs}}\left(V,p_{\mathrm{obs}}\right)$ denoting the distance between the node *V* and the nearest obstacle $p_{\mathrm{obs}}$. The attractive term is deliberately omitted for the following reason. During roadmap construction there is no designated target to approach: the roadmap must uniformly cover the whole free space in order to serve as a sparse topological backbone for pairwise frontier path planning. An attractive field pointing to any fixed location would bias the node distribution toward that location and destroy the uniformity of the coverage, whereas the repulsive term alone is sufficient to push nodes away from obstacles toward the medial regions of the free space, which is exactly the backbone structure required. The gradient of the potential field is then computed, and the node position is updated along the negative gradient direction, i.e., the direction in which the repulsive potential decreases:

(3) $$V^{\prime }=V-c_{s}\frac{\partial U(V)}{\partial V},$$

where $c_{s}$ is a constant employed to regulate the extent of position optimization. Since $\nabla U=-e^{-D_{\mathrm{obs}}}\nabla D_{\mathrm{obs}}$ and $\nabla D_{\mathrm{obs}}$ points away from the nearest obstacle, the displacement $-c_{s}\nabla U$ always repels the node from obstacles, and its magnitude $c_{s}e^{-D_{\mathrm{obs}}}$ decays exponentially with the obstacle distance, so nodes deep in open space are barely moved while nodes near obstacles are pushed toward spacious areas. Considering that $c_{s}$ cannot be uniquely determined, a multi-step iterative correction strategy is adopted. This strategy performs numerous short-distance position corrections based on Equation (3) to prevent a single long-distance position correction from bringing the position too near other obstacles.

As shown in Figure 3, node *V* is initially close to the right obstacle. Following a single correction, as shown by the blue point, its position is pushed away from the right obstacle; after the second correction, as shown by the yellow point, it settles in the center of the road. In contrast, the remaining nodes are situated in non-constricted areas. Thus, each correction only steers them towards spacious areas.

After obtaining the corrected nodes, calculating the nearest node to the frontier becomes expensive as the frontiers do not coincide with the nodes. To tackle this issue, we merge the frontiers into the nodes and then employ the Delaunay triangulation algorithm [30] to generate road edges *E*.

## 5. Traversal Path Planning

Obtaining frontiers is unquestionably valuable for exploration, and traversing the frontiers is undoubtedly the optimal solution. However, before addressing the TSP for frontiers, it is crucial to determine the distances between each pair of frontiers. Merely considering the Euclidean distance between frontiers is insufficient for a comprehensive solution. Our objective is to achieve autonomous exploration. Therefore, we consider both path cost and information gain when conducting path planning.

### 5.1. Information Entropy-Based Path Planning

We design an IDijkstra algorithm for path planning on the road map G(V,E) which can simultaneously plan for multiple goals. The IDijkstra algorithm offers several improvements over the traditional Dijkstra algorithm.

Firstly, during the search process, the cost calculation considers both the path cost and the information gain.

(4) $$f_{V}=g_{V}+\left[1+H_{IE}\left(V_{c},V_{n}\right)\right]\cdot D_{V}\left(V_{c},V_{n}\right),$$

where $g_{V}$ represents the cumulative distance from the starting node to the current node $V_{c}$, $D_{V}\left(V_{c},V_{n}\right)$ represents the distance between $V_{c}$ and its neighboring node $V_{n}$, and $H_{IE}\left(V_{c},V_{n}\right)$ is the calculation function for the information entropy coefficient. This coefficient is used to search for paths that are more inclined toward unknown areas, and its calculation expression is defined as follows:

(5) $$H_{IE}\left(V_{c},V_{n}\right)=\min\left[\exp\left(-\frac{D_{\mathrm{free}}\left(V_{c}\right)}{c_{V}}\right),\exp\left(-\frac{D_{\mathrm{free}}\left(V_{n}\right)}{c_{V}}\right)\right],$$

where $D_{\mathrm{free}}\left(V\right)$ is used to calculate the shortest distance from node *V* to the known space in the grid map OGM. As stated in Equation (5), it can be inferred that when either node $V_{c}$ or $V_{n}$ is located far from the known space, the corresponding node exhibits a higher information gain, indicating the necessity for the robot to explore that particular area. Consequently, the information entropy coefficient of such a node becomes smaller. This method offers a lower computational cost than directly using ray casting to calculate the information gain for each edge accurately.

The form of Equation (4) is chosen deliberately. Since $H_{IE}\left(V_{c},V_{n}\right)$ is a dimensionless coefficient in $\left(0,1\right]$, the factor $\left[1+H_{IE}\left(V_{c},V_{n}\right)\right]$ is an inflation multiplier in $\left(1,2\right]$ that biases the edge cost toward unknown regions while preserving the semantics of a scaled path length. Three properties follow. First, the edge cost is always strictly positive, which maintains the nonnegativity assumption required by Dijkstra-type searches. Second, the path-length term always dominates the cost, so the planned path never degenerates into a pure pursuit of entropy and remains a valid geometric path. Third, the influence of information is bounded—it can at most halve the relative cost of an edge (i.e., an edge in a highly unknown region is at most twice as attractive relative to its length)—which keeps the search stable across maps with different entropy scales. In contrast, a purely multiplicative form $H_{IE}\cdot D_{V}$ would drive the edge cost toward zero in highly unknown regions and could create zero-cost cycles that break Dijkstra’s optimality guarantee, whereas a subtractive reward form $g_{V}+D_{V}-H_{IE}$ mixes incompatible units (entropy versus distance) and may even produce negative edge costs. The chosen additive-plus-multiplicative form therefore provides a principled trade-off between information bias and metric consistency.

Furthermore, the termination condition for the planning phase expands from a single target node to a set of target nodes. The end of the search is marked by finding a global path that reaches all the frontiers. The complete procedure of IDijkstra is presented in Algorithm 1.
**Algorithm 1** IDijkstra**Require:** Roadmap G(V,E), Robot Pose $p_{\mathrm{bot}}$, Frontier Set $P_{f}$**Ensure:** Global path set $P_{\mathrm{path}}$, updated roadmap $G^{\prime }\left(V,E^{\prime }\right)$  1:Initialize open set $O_{\mathrm{set}}$, closed set $C_{\mathrm{set}}$;  2:Add robot current position to $O_{\mathrm{set}}$: $O_{\mathrm{set}}\leftarrow O_{\mathrm{set}}\cup V_{S}$;  3:**while** $O_{\mathrm{set}}\neq \emptyset$ **do**  4:      Pop node $V_{C}$ with minimum cost from $O_{\mathrm{set}}$;  5:      Add $V_{C}$ to $C_{\mathrm{set}}$;  6:      **if** $V_{C}$ is in $P_{f}$ **then**  7:             Backtrack path from $V_{C}$ and store to $P_{\mathrm{path}}$;  8:             **if** all frontiers in $P_{f}$ have been settled **then**  9:                   **return**;10:             **end if**11:      **end if**12:      **for** each neighbor $V_{n}$ of $V_{C}$ in G(V,E) **do**13:            **if** edge *E* collides with obstacle **then**14:                  Roadmap maintenance: $G^{\prime }\left(V,E^{\prime }\right)\leftarrow G\left(V,E\right)$;15:                  **continue**;16:            **end if**17:            **if** $V_{n}$ is in $C_{\mathrm{set}}$ **then**18:                   **continue**;19:            **end if**20:            compute cost $f_{V\mathrm{temp}}$ using Equation (4);21:            **if** $V_{n}$ is in $O_{\mathrm{set}}$ **then**22:                   **if** $f_{V\mathrm{temp}}<f_{V}$ **then**23:                        update $V_{n}$: $f_{V}\leftarrow f_{V\mathrm{temp}}$;24:                   **end if**25:             **else**26:                   Add $V_{n}$ to $O_{\mathrm{set}}$;27:             **end if**28:       **end for**29:**end while**30:Remove unsettled (unreachable) frontiers from $P_{f}$;

To be precise about the termination and the computational cost, we state the following. Within one run, nodes are settled (popped from the open set with their minimal cost finalized) in ascending order of $f_{V}$, exactly as in standard Dijkstra. The run terminates early as soon as the *last* frontier in $P_{f}$ has been settled: at that moment, optimal global paths from the source to *all* frontiers have been obtained simultaneously, because Dijkstra settles nodes in nondecreasing cost order and the paths to all other frontiers have costs no larger than that of the last settled frontier. In other words, one single-source multi-target run replaces $\left|P_{f}\right|$ independent single-goal searches. If the open set empties before all frontiers are settled, the remaining frontiers are unreachable from the source and are discarded.

To obtain the pairwise distances required for TSP solving, IDijkstra is executed once from the robot position $p_{\mathrm{bot}}$ and then once from each frontier, i.e., $\left|P_{f}\right|+1$ runs in total, which yields the global paths from the robot to every frontier and between every pair of frontiers. Each run costs $O\left(\left|E\right|+\left|V\right|\log\left|V\right|\right)$ with a binary-heap implementation, so the overall complexity is $O\left(\left(\left|P_{f}\right|+1\right)\cdot \left(\left|E\right|+\left|V\right|\log\left|V\right|\right)\right)$, i.e., linear rather than quadratic in the number of frontiers, since the multi-target early termination removes the need for a separate run per frontier pair. This cost is affordable in practice for three reasons. First, the roadmap is a sparse backbone of the environment: $\left|V\right|$ and $\left|E\right|$ are orders of magnitude smaller than the number of grid cells, and each node has only a constant number of Delaunay neighbors. Second, the number of frontiers $\left|P_{f}\right|$ remains moderate (a few tens in the environments of Section 7), and the early termination rarely requires expanding the entire graph. Third, the computation can be further accelerated by caching the pairwise distances obtained in a planning step and re-computing only the paths whose edges were invalidated by newly detected obstacles, which keeps the per-step cost approximately proportional to the number of frontier changes rather than to $\left|P_{f}\right|$.

### 5.2. Adaptive Iterative Cyclic Crossover COOT

After obtaining the distance between frontiers, the improved ACXCOOT algorithm is used to solve the optimal traversal order $T_{fT}$ of the frontiers, commencing from position $p_{\mathrm{bot}}$ of the robot. The detailed steps can be referenced in Algorithm 2, whose time complexity is $O\left(n_{pf}\cdot {\mathrm{Iter}}_{\mathrm{num}}\right)$, where ${\mathrm{Iter}}_{\mathrm{num}}$ is adaptively determined by Equation (6). This adaptive mechanism ensures that the iteration count scales reasonably with the number of frontiers, prioritizing real-time performance over exhaustive optimization when frontier density is high. For more details about the COOT algorithm, please refer to [31].
**Algorithm 2** ACXCOOT.**Require:** Global Path $P_{\mathrm{path}}$**Ensure:** Frontier Traverse Order $T_{fT}$  1:${\mathrm{pop}}_{\mathrm{size}}$, $l_{n}$, ${\mathrm{tol}}_{\mathrm{thr}}$, ${\mathrm{Iter}}_{\max}$← Parameters Initialization();  2:${\mathrm{Iter}}_{\mathrm{num}}=b_{1}\cdot e^{b_{2}\cdot n_{pf}}\;\left({\mathrm{Iter}}_{\mathrm{num}}\leq {\mathrm{Iter}}_{\max}\right)$;  3:${\mathrm{coot}}_{\mathrm{pop}}$← Traversal Order Random Initialization $\left({\mathrm{pop}}_{\mathrm{size}}\right)$;  4:fitness← Fitness Calculation $\left({\mathrm{coot}}_{\mathrm{pop}},P_{\mathrm{path}}\right)$;  5:${\mathrm{coot}}_{\mathrm{best}},{\mathrm{coot}}_{g},{\mathrm{coot}}_{o}$← Classification $\left({\mathrm{coot}}_{\mathrm{pop}},fitness,l_{n}\right)$;  6:${\mathrm{Arr}}_{\mathrm{tol}}$← Tolerance Initialization $\left({\mathrm{tol}}_{\mathrm{thr}},{\mathrm{coot}}_{g}\right)$;  7:t←0;  8:**while** $t<{\mathrm{Iter}}_{\mathrm{num}}$ **do**  9:      ${\mathrm{coot}}_{\mathrm{best}},{\mathrm{coot}}_{g},{\mathrm{coot}}_{o}$← Reclassification $\left({\mathrm{coot}}_{g},{\mathrm{coot}}_{o},{\mathrm{Arr}}_{\mathrm{tol}},fitness\right)$;10:      ${\mathrm{coot}}_{o}$← Traversal Order Regeneration $\left({\mathrm{coot}}_{o}\right)$;11:      ${\mathrm{coot}}_{g},{\mathrm{Arr}}_{\mathrm{tol}}$← Leaders Position Update $\left({\mathrm{coot}}_{\mathrm{best}},{\mathrm{coot}}_{g},{\mathrm{Arr}}_{\mathrm{tol}}\right)$;12:      fitness← Fitness Calculation $\left({\mathrm{coot}}_{g},{\mathrm{coot}}_{o},P_{\mathrm{path}}\right)$;13:      ${\mathrm{coot}}_{\mathrm{best}}$← Update $\left({\mathrm{coot}}_{\mathrm{best}},{\mathrm{coot}}_{g},{\mathrm{coot}}_{o},fitness\right)$;14:      t←t+1;15:**end while**16:**return** $T_{fT}\leftarrow {\mathrm{coot}}_{\mathrm{best}}$

The ACXCOOT algorithm classifies the population into three categories: the best individual ${\mathrm{coot}}_{\mathrm{best}}$, group leaders ${\mathrm{coot}}_{g}$, and ordinary individuals ${\mathrm{coot}}_{o}$. Ordinary individuals explore by generating traversal orders for the frontiers randomly, preventing the algorithm from being trapped in local optima. Group leaders use the cyclic crossover operator to optimize their positions toward the best individual. The cyclic crossover operator does not require conflict detection compared to other operators, which reduces the time complexity of the algorithm. Let gene sequence A denote a group leader and gene sequence B denote the best individual. After applying the cyclic crossover operator, the group leader becomes C, while the best individual is reserved via elitism and remains B.

*Step A*: Randomly select a gene segment at position *k* from gene sequence A.

*Step B*: Copy the gene segment at position *k* from A to the corresponding position in sequence C.

*Step C*: In gene sequence B, locate the gene segment at the same position *k*.

*Step D*: In gene sequence A, find the position $k^{\prime }$ where the gene element equals that found in Step C. If $k^{\prime }\neq k$, repeat Steps B–D using position $k^{\prime }$; otherwise, proceed to Step E.

*Step E*: Fill all remaining vacant positions in sequence C with the corresponding genes from sequence B.

It should be noted that the group leaders possess a tolerance attribute that strengthens the optimization capability of ACXCOOT. Once the optimization attempts made by a group leader exceed the threshold ${\mathrm{tol}}_{\mathrm{thr}}$, it is demoted to an ordinary individual. Subsequently, a new group leader is selected from the ordinary individuals. This expands the optimization range of ACXCOOT. The rationale of this demotion mechanism is stagnation control. A leader that has failed to improve its fitness for ${\mathrm{tol}}_{\mathrm{thr}}$ consecutive generations is, with high probability, trapped in a local basin of the solution landscape; continuing to refine it would only waste evaluations. Replacing it with a freshly regenerated ordinary individual re-injects diversity into the population and restores global exploration pressure, which prevents premature convergence to local optima. This is the same principle as the abandonment of exhausted food sources by scout bees in the artificial bee colony algorithm [32], and it achieves the exploration–exploitation re-balancing at a bounded cost of at most ${\mathrm{tol}}_{\mathrm{thr}}$ wasted evaluations per demoted leader.

To meet real-time requirements, an iterative adaptive mechanism is designed to find the optimal solution quickly. This is achieved by offline fitting the function expression of the number of frontiers and the iteration count of the algorithm:

(6) $${\mathrm{Iter}}_{\mathrm{num}}=b_{1}\cdot e^{b_{2}\cdot n_{pf}},$$

where $b_{i},i\in \left\{1,2\right\}$ represents the fitting coefficients and $n_{pf}$ is the number of frontiers. The coefficients were determined by offline calibration as follows. For frontier sets of different sizes, ACXCOOT was run with a generous fixed iteration budget of ${\mathrm{Iter}}_{\max}$, and for each size $n_{pf}$ we recorded the number of iterations after which the best fitness no longer improved by more than a relative gap of 1% until the end of the run; least-squares fitting of the exponential model in Equation (6) to these saturation counts yielded $b_{1}=0.45$ and $b_{2}=0.93$. Regarding sensitivity, Equation (6) is monotone in $n_{pf}$ and is clamped by ${\mathrm{Iter}}_{\max}$, which guarantees a bounded runtime for any number of frontiers: a perturbation of $b_{1}$ merely rescales the iteration count by the same ratio, whereas the response to $b_{2}$ grows with $n_{pf}$, which is precisely why the cap ${\mathrm{Iter}}_{\max}$ is indispensable. Because the solution quality plateaus around the saturation count—as reflected by the small standard deviations in the path costs in the comparative experiments reported in Section 7—moderate deviations of the fitted coefficients from their nominal values do not measurably affect the final path cost, only the number of redundant iterations. When there are many frontiers, finding the optimal solution with many iterations becomes difficult.

Thus, it is necessary to prioritize the real-time performance of the algorithm. A maximum iteration limit of ${\mathrm{Iter}}_{\mathrm{num}}$ is also set with the requirement that ${\mathrm{Iter}}_{\mathrm{num}}\leq {\mathrm{Iter}}_{\max}$.

### 5.3. Distance Orientation Evaluation

The traversal order of the frontiers actually forms an undirected closed-loop graph. If the robot follows this order, it may traverse the same area, increasing the path cost unnecessarily. As shown in Figure 4a,b, the traversal sequences of the frontiers generated in consecutive exploration steps are presented.When the robot visits the frontiers in reverse order, it travels from node A to node F during the first exploration. However, during the second exploration, if the frontiers are visited sequentially, the robot travels from node F to node B, passing through node A again, resulting in duplicated path segments.

To address this issue, a reference trajectory (indicated by the red arrow in Figure 4) is utilized to prevent path repetition. The current traversal direction is evaluated using cosine similarity, and a decision variable for path cost is incorporated. If the path cost to reach one frontier is much lower than the path cost to reach another, the primary decision variable should be the path cost. The evaluation function is defined as follows:

(7) $$L=\chi_{1}\frac{\cos\mathrm{sim}\left(T_{\mathrm{ref}},T_{i}\right)}{\sum_{j=1}^{2}\cos\mathrm{sim}\left(T_{\mathrm{ref}},T_{j}\right)}-\chi_{2}\frac{D_{L}\left(T_{i}\right)}{\sum_{j=1}^{2}D_{L}\left(T_{j}\right)},$$

where cossim(·) represents the cosine similarity calculation function, and $T_{\mathrm{ref}}$ is the reference trajectory, $T_{i},i\in \left\{1,2\right\}$ is the current driving trajectory. $D_{L}\left(\cdot \right)$ is used to calculate the distance of the driving trajectory, while $\chi_{i},i\in \left\{1,2\right\}$ represents the weights with the constraint $\chi_{1}+\chi_{2}=1$. In Equation (7), we select the frontier with the maximum *L* value as the NBV for environmental exploration.

## 6. Path Tracking and Update

After selecting the NBV, the APG-SAC drives the robot to explore the environment according to the global path planning in Section 5. However, as the environment exploration proceeds, the planned path inevitably intersects with obstacles, so real-time path updates are necessary. Additionally, updating the NBV can reduce path costs.

### 6.1. APG-SAC Motion Control

In the APG-SAC algorithm, the state input is a triplet $s_{t}=\left[s_{t}^{\mathrm{laser}},s_{t}^{\mathrm{goal}},s_{t}^{\mathrm{speed}}\right]$, where $s_{t}^{\mathrm{goal}}=\left[d_{t},\theta_{t}\right]$ represents the relative distance and orientation between the position of the robot and the NBV at time *t*. $s_{t}^{\mathrm{speed}}=\left[v_{t-1},\omega_{t-1}\right]$ represents the linear velocity and angular velocity of the robot at time t−1. $s_{t}^{\mathrm{laser}}$ represents the laser beam at time *t* that has been processed by the APG module, as shown in Figure 5. Assuming the detection range of the laser is $\varphi \in \left[\varphi_{\min},\varphi_{\max}\right]$ with an angular resolution of $res_{s}$, the total number of laser beams is $n_{s}=\left(\varphi_{\max}-\varphi_{\min}\right)/res_{s}$. The laser detection area is divided into $A_{s}$ sector regions. Since the number of beams per sector must be an integer, the first $n_{s}\;\mathrm{mod}\;A_{s}$ sectors are assigned $\left\lceil n_{s}/A_{s}\right\rceil$ consecutive beams each and the remaining sectors $\left\lfloor n_{s}/A_{s}\right\rfloor$ beams each, so that adjacent sectors differ by at most one beam. The minimum value is selected from each sector region, resulting in $A_{s}$ beams. These beams are then combined to form the $s_{t}^{\mathrm{laser}}$.

The action output is a tuple $a_{t}=\left[v_{t},\omega_{t}\right]$, representing the robot’s linear velocity and angular velocity at time *t*. These values must adhere to the robot’s kinematic constraints, namely, $v_{t}\in \left[v_{\min},v_{\max}\right]$ and $\omega_{t}\in \left[-\omega_{\max},\omega_{\max}\right]$.

Regarding the reward function, we solely use the APG-SAC for motion control. Consequently, the design of the reward function should be related to the position of reaching NBV $r_{g}$, colliding with obstacles $r_{c}$, and the smoothness of robot motion $r_{\omega }$:

(8) $$r_{t}=r_{g}+r_{c}+r_{\omega }.$$

$r_{g}$ comprises a positive reward of +10 for reaching NBV or 0 otherwise. Similarly, $r_{c}$ is a negative reward of −10 for colliding with obstacles, or 0 otherwise. $r_{\omega }=-0.05\times {\left(\omega_{t-1}-\omega_{t}\right)}^{2}$ means that significant changes in angular velocity between adjacent moments are penalized to ensure smooth travel. It should be noted that due to the floating-point nature of the $\omega_{t}$, the relationship $r_{\omega }\leq 0$ always holds. The smoothness penalty $r_{\omega }$ encourages steady motion, while reaching the NBV ($r_{g}$) and avoiding collisions ($r_{c}$) constitute the primary behavioral objectives. Although the reward function contains no explicit speed term, it still favors prompt arrival: $r_{g}$ is a terminal reward that, under the standard discounted return, is worth more the sooner it is collected, so the optimal policy reaches the NBV in fewer time steps for the same safety and smoothness level; the penalty $r_{\omega }$ then ensures that this prompt arrival is realized through steady, uninterrupted motion rather than through aggressive swerving. Hence, the lower exploration time of ETPP reported in Section 7 results from two complementary factors: (i) the shorter planned paths produced by the global traversal planning, and (ii) a higher average execution speed maintained by smoother motion with fewer deceleration events. In this sense, the “more aggressive” behavior of the APG-SAC controller mentioned in Section 7 refers to decisive, uninterrupted motion toward the goal, not to a reward that explicitly maximizes speed.

The training network comprises three layers of fully connected neurons, each containing 512 nodes. We do not need to design an overly complicated network to achieve the ideal effect.

### 6.2. Path Update

As the environment is continuously explored, the planned path inevitably encounters obstacles. Therefore, real-time path updates become essential. A* algorithm is used for planning on the road map *G*. Since the nodes are uniformly scattered with spacing $c_{V}$ and the Delaunay triangulation connects neighboring nodes, *G* locally approximates an equilateral triangular lattice with side length $c_{V}$. To obtain a provably admissible heuristic for such a lattice, each node $V\left(x,y\right)$ is associated with the nearest vertex $\left(i,j\right)$ of the ideal lattice, with $i=\mathrm{round}\left(x/c_{V}\right)$ and $j=\mathrm{round}\left(y/\left(c_{V}\sin\left(\pi /3\right)\right)\right)$. The minimum number of unit lattice steps between two lattice vertices admits the closed-form distance formula for hexagonal grids [33], from which the heuristic is derived as follows:

(9) $$\left\{\begin{array}{c} \Delta i=i_{g}-i_{c},\;\Delta j=j_{g}-j_{c},\\ \Delta x_{c}=\Delta i-\frac{\left(j_{g}-j_{g}\;\mathrm{mod}\;2\right)-\left(j_{c}-j_{c}\;\mathrm{mod}\;2\right)}{2},\\ h_{A}=\frac{\sqrt{3}}{2}\cdot \frac{c_{V}}{2}\left(\left|\Delta x_{c}\right|+\left|\Delta x_{c}+\Delta j\right|+\left|\Delta j\right|\right), \end{array}\right.$$

where $\left(i_{c},j_{c}\right)$ and $\left(i_{g},j_{g}\right)$ denote the lattice indices of the current node $V_{c}\left(x_{c},y_{c}\right)$ and the NBV $V_{g}\left(x_{g},y_{g}\right)$, respectively, and mod denotes the remainder operation. The first two lines convert the row–column offset into axial coordinates, and the third line evaluates the hexagonal distance [33].

The admissibility of $h_{A}$ is established as follows. On the ideal equilateral lattice, the term $\left(c_{V}/2\right)\left(\left|\Delta x_{c}\right|+\left|\Delta x_{c}+\Delta j\right|+\left|\Delta j\right|\right)$ equals the exact shortest-path distance between the two lattice vertices, because every unit edge of the lattice has length $c_{V}$ [33]. Moreover, for any pair of lattice vertices, the Euclidean distance is at least $\sqrt{3}/2$ times this lattice distance, which is why the scaling factor $\sqrt{3}/2$ is introduced. Since every roadmap edge is a straight segment, the true remaining cost from $V_{c}$ to $V_{g}$ is never smaller than their Euclidean distance, and therefore $h_{A}$ never overestimates the true cost. Consequently, A* with $f_{A}=g_{A}+h_{A}$ returns optimal paths on the roadmap [34], while the residual node displacements introduced by the potential-field correction are small relative to $c_{V}$ and are absorbed by the $\sqrt{3}/2$ margin. Thus, the cost calculation is shown in Equation (10):

(10) $$f_{A}=g_{A}+h_{A},$$

where $g_{A}$ is the distance from the starting node to $V_{c}$.

### 6.3. Target Update

When the exploration goal of the robot is located in a corner, the robot does not need to reach NBV to complete the exploration of the environment. For this situation, we use mutual information to evaluate the information gain within the target area $A_{\mathrm{tar}}$ and determine whether the NBV needs to be updated. We use the ray casting method to calculate the information entropy of $A_{\mathrm{tar}}$ and compare it with the expected information entropy, as shown in Equation (11):

(11) $$I=H\left(A_{\mathrm{tar}}\right)-H\left(A_{\mathrm{tar}}|T_{\mathrm{tar}}\right),$$

where $H\left(A_{\mathrm{tar}}\right)$ represents the map entropy of $A_{\mathrm{tar}}$, and $H\left(A_{\mathrm{tar}}|T_{\mathrm{tar}}\right)$ represents the map entropy of $A_{\mathrm{tar}}$ after traversing the path $T_{\mathrm{tar}}$ to the NBV. The calculation formula for map entropy is expressed as follows:

(12) $$H\left(A_{\mathrm{tar}}\right)=-\sum_{i=1}^{N}p\left(m_{i}\right)\log\left[p\left(m_{i}\right)\right],$$

where $m_{i}$ represents the *i*-th grid of $A_{\mathrm{tar}}$. In the grid map, when a grid $m_{i}$ is known, $p\left(m_{i}\right)=0$, and when it is unknown, $p\left(m_{i}\right)=0.5$. From Equation (11), we can analyze $H\left(A_{\mathrm{tar}}|T_{\mathrm{tar}}\right)=0$, which means that $A_{\mathrm{tar}}$ solely comprises known grids after the robot reaches the NBV. Therefore, Equation (11) can be rewritten as $I=H\left(A_{\mathrm{tar}}\right)$. To facilitate the judgment of information gain in the $A_{\mathrm{tar}}$, we normalize it:

(13) $$I=\frac{H(A_{\mathrm{tar}})}{H_{\max}\left(A_{\mathrm{tar}}\right)},$$

where $H_{\max}\left(A_{\mathrm{tar}}\right)$ represents the maximum map entropy when all grids within $A_{\mathrm{tar}}$ are unknown. When $I\leq I_{\mathrm{thr}}$ is met, it is determined that there is no further exploration value within $A_{\mathrm{tar}}$, and a new NBV is selected. The threshold value $I_{\mathrm{thr}}$ is predetermined.

## 7. Experiments and Results

### 7.1. Environment Setup

To assess the performance of ETPP, we conduct experiments in ten simulation environments of varying sizes and structures as shown in Figure 6. The sizes of these environments range from 10×8 m^2^ to 30×30 m^2^. Five experiments are performed for each method in each simulation environment to minimize the influence of randomness. The compared methods include greedy exploration (Greedy) [8], random exploration (Random), nearest frontier exploration (Nearest) [14], and exploration based on multiple rapidly exploring random trees (Multi-RRT) [13].

All four comparison methods employed are categorized as frontier-based methods. However, they vary in terms of frontier generation and NBV strategy. The Greedy follows the classical frontier-based exploration scheme of [8]: frontiers are detected by breadth-first search over the grid map, and the one offering the highest information gain is selected as the NBV. The Nearest implements the nearest-frontier strategy evaluated in [14], in which the NBV is the frontier with the lowest path cost from the robot. The Multi-RRT follows the multi-RRT exploration framework of [13]: multiple rapidly exploring random trees are grown to detect frontiers in parallel with robot motion, and the NBV is chosen through a comprehensive evaluation that combines path cost and information gain. The Random is a standard stochastic baseline that shares the RRT-based frontier detection of Multi-RRT but uniformly samples the NBV among all detected frontiers; it is included to quantify the benefit of informed NBV selection.

All the experiments are conducted on a computer with AMD R7-5800H CPU and 16 GB of memory. The parameters of the ETPP framework are shown in Table 1. Among them, ${\mathrm{pop}}_{\mathrm{size}}$, $l_{n}$, ${\mathrm{tol}}_{\mathrm{thr}}$ and ${\mathrm{Iter}}_{\max}$ are the key parameters that affect the efficiency of the algorithm and need to be adjusted by many experiments according to the actual situation.

### 7.2. Simulation Experiments

#### 7.2.1. Autonomous Exploration Process

The process of autonomous exploration in unknown environment is shown in Figure 7. The environment is built by simulating the mall floor.

The eight diagrams in Figure 7 are obtained by real-time interception during the robot exploration. Figure 7 illustrates that the road map generation method can effectively highlight the backbone information of free space in an environment. Although some road edges cross obstacles, the paths generated bypass them. The robot follows the paths and generates new frontiers continuously during the exploration.

Figure 7f,g depict that the generated path is more inclined to drive in unknown areas when the environmental information is uncertain. The blue curve in Figure 7f demonstrates this. The blue curve exhibits a higher information gain despite the red curve having a shorter path length. This further supports the efficacy of our IDijkstra path planning algorithm. As the exploration of the environment progresses, the planned path overlaps with obstacles, and path updates are made to guarantee that the robot can safely reach the destination, as presented in Figure 7h. Once the entire environment has been explored, the robot stops.

#### 7.2.2. Path Cost and Time Cost

We analyze the performance of five different methods in different simulated environments. Figure 8 shows the exploration trajectories close to the average level of each method. Each graph’s title displays the path cost of a method in the respective environment, whereas the bottom right corner depicts the method name.

Figure 8 demonstrates that robots can thoroughly explore the environment in some relatively enclosed spaces without fully entering and reselecting NBV using the target point update mechanism, as observed in Env 2. It should be noted that if the sensors equipped on the robot cannot entirely detect the environmental space, the robot must enter the area for exploration, as seen in Env 6. Furthermore, in Figure 8, the ETPP and Greedy driving trajectories are mostly located in the center of the road, particularly noticeable in Env 1. ETPP generates frontiers that are entirely at the center of the road. Although Greedy generates frontiers near the obstacles and in the center of the road, the target selection strategy directs the robot towards the more spacious areas. Nonetheless, the Greedy exhibits certain shortcomings, as in Env 2, where it guides the robot to drive on the main road first, followed by exploration in each room, leading to repeated driving in some areas. In contrast, ETPP explores the main road concurrently with room-by-room exploration, significantly reducing the path cost.

The Nearest selects the NBV based on the shortest distance to the frontier, leading to frequent acceleration and deceleration problems for the robot and increased risk due to the selection of NBVs that are closer to obstacles. Figure 8 illustrates that this method often results in aimless wandering, which increases path cost and is ineffective. The Random method randomly selects NBVs and is the least effective method. Among the four algorithms aside from ETPP, the Multi-RRT algorithm shows the most comprehensive performance despite sharing issues with the Nearest.

In order to better evaluate all methods, we design two large environments, Env 9 and Env 10. Env 9 is a highly challenging environment with many forks, and based on the experimental results, the ETPP algorithm performed as expected. In Env 10, the most reasonable path is undoubtedly for the robot to explore another road at each fork after passing through the main road. However, the robot deployed with the ETPP does not follow this path. The reason is that the limited sensor range makes it impossible to plan the optimal path. Although the ACXCOOT can generate the optimal traverse order of the frontiers, it is only reliable at the current moment and does not guarantee global optimality.

Figure 9 shows the variation curves of map entropy versus exploration time for multiple methods in a single experiment. Regarding exploration speed and effectiveness, the ETPP outperforms the other four methods. In Env 2, ETPP and Multi-RRT take roughly the same amount of time, but the map entropy of ETPP decreases faster. In Env 4, the method based on Nearest has a significantly longer exploration time and does not significantly reduce the map entropy. This is mainly caused by unexplored environmental regions that require substantial travel time for the robot to reach. During this travel, the robot passes through known spaces.

Figure 10 depicts the time and path cost of five methods across all simulated environments. ETPP exhibits the lowest time and path cost in all environments, indicating that ETPP applies to various environments. Although the path cost of the Random tends to be higher, its time cost remains lower than that of the Greedy in some environments. Two potential reasons exist for this phenomenon. Firstly, the Greedy select an NBV based on information gain, but the NBV may be located far from the robot’s current position. Secondly, the Greedy takes more time to generate frontiers. The Nearest outperforms the Greedy, suggesting that exploring based only on path cost is more effective than considering only information gain when the exploration termination criteria are identical. In Env 5, where only a single passable path exists, ETPP demonstrates similar path cost to other methods but outperforms significantly in exploration time, which indicates that our motion controller maintains a higher average speed through smoother, uninterrupted motion (Section 6).

We present the average path cost, time cost, and percentage improvement in the ETPP compared to other methods in Table 2 and Table 3 for quantitative analysis. The best data for each environment are highlighted in bold for easy reference. Table 2 shows that ETPP achieves an average reduction in path cost of 15.21 m (15.95%) and in time cost of 96.73 s (21.04%) compared to the Multi-RRT. Even in Env 5, ETPP decreases the path cost and time cost by 2.62 m (9.10%) and 15.51 s (12.62%) compared with the Multi-RRT, and this is entirely due to the smoother driving of the APG-SAC motion planning algorithm.

#### 7.2.3. Real-Time Performance

Figure 11 compares the real-time performance of the proposed ACXCOOT algorithm with other intelligent optimization algorithms. The histogram shows the total executions of the different algorithms in five experiments in each environment. On the other hand, the line chart shows the average single calculation time of the algorithms. ACXCOOT reduces computational time by an average of 0.77 s (46.10%) compared to COOT, thereby improving real-time performance. It is noteworthy that ACXCOOT has the smallest time consumption among the five different intelligent optimization algorithms.

To demonstrate that the ACXCOOT can improve the real-time performance while maintaining solution accuracy, we record the path cost generated by using ACXCOOT and other intelligent optimization algorithms, as shown in Figure 12. From an overall perspective, the ACXCOOT has the smallest path cost and standard deviation, which indicates that it is more stable, and least affected by the environment.

#### 7.2.4. Simulation Performance Under Different Conditions

We compare ETPP and Multi-RRT, the top-performing approach among the other methods. We compare both methods under different algorithm parameters and hardware conditions, as shown in Table 4. The evaluation is limited to challenging large-scale Env 9 and Env 10.

Figure 13 shows that when the detection radius of the laser $d_{m}$ is increased, there is a significant decrease in the path cost and time cost of robot exploration. Interestingly, when the parameter $c_{V}$ is increased, ETPP-III has lower path and time costs than ETPP-II in Env 9, but they increase in Env 10. This is because a larger $c_{V}$ value makes the path planned by IDijkstra more tortuous, resulting in increased path and time costs. Overall, ETPP outperforms the other methods.

### 7.3. Real-World Experiments

To further validate the ETPP, we conduct tests in four distinct real-world scenarios. Env 1 and Env 2 are constructed by placing numerous random obstacles. Env 3 represents an indoor office environment, while Env 4 simulates an office floor, as depicted in Figure 14. The mapping effect and travel trajectory are visualized in the bottom right corner of the figure.

In Env 1, robot exploration is based on the frontier $p_{f2}$. This is due to the robot’s sensors not reaching the maximum detection range in this environment, resulting in an inability to generate the frontier $p_{f1}$. In Env 2, robot exploration is based on frontier $p_{f1}$. Notably, this environment only features surrounding walls without any additional large obstacles. The robot deployed with ETPP starts from the bottom left corner and explores the entire environment by circling around it.

Table 5 illustrates that the robot’s driving speed is lower in Env 3 due to narrower roads than in Env 1. In Env 3, we place the robot at an intersection. Due to the limited range of the sensors, it is understandable that the robot initiates exploration from any direction. Fortunately, it chooses the side with a shorter path cost. Furthermore, the robot effectively navigates narrow passages no wider than twice its size within this environment.

Env 4 is intentionally chosen as a large-scale environment. Due to the limitations of localization accuracy, complete exploration in this environment cannot be achieved even with the cartographer algorithm. However, from the exploration trajectory, we can observe that the robot always maintains a position in the middle of the road. This indicates that the designed APG-SAC motion controller can perform well even in real-world scenarios, with the network parameters solely trained in simulation environments.

This experiment also exposes a limitation of the current framework. ETPP, like most frontier-based systems, relies on sufficiently accurate localization: the frontier positions, the roadmap, and the termination condition are all maintained in the global map frame. In large, feature-sparse environments where scan matching and loop closure degrade, the drifting global pose distorts the occupancy grid and shifts the generated frontiers, so that the exploration may terminate prematurely or revisit previously covered regions. In such situations the robot still does not collide, because the APG-SAC controller acts on local sensor observations and keeps the robot in the middle of the corridors even under mild pose drift, but complete coverage can no longer be guaranteed. To make the framework robust under poor localization, the following practical measures can be adopted: (i) monitoring localization quality, e.g., the scan-matching score and the pose-graph residual, and triggering re-localization or pausing exploration when it falls below a threshold; (ii) fusing complementary sensors such as IMU, wheel odometry, and, where available, visual or UWB anchors to bound the pose drift; and (iii) pruning frontiers conservatively in map regions with high uncertainty. Integrating such a localization-quality gate into ETPP is part of our future work.

## 8. Conclusions

In this paper, we propose ETPP, a traversal path planning framework that enables the autonomous exploration of unknown environments. The method presented in this paper has the following characteristics: a hybrid strategy is designed to generate frontiers to ensure comprehensive exploration of the environment, and a potential field is used to construct a road map for accelerated path planning. This road map can effectively describe the backbone information in the environment. Unlike other methods, ETPP does not require time-consuming calculation of frontier information gain. Instead, all frontiers are considered to require exploration. Therefore, an ACXCOOT algorithm is adopted to solve the frontier TSP problem, reducing path costs. In addition, a distance–orientation evaluation function is designed to select NBV. Finally, an APG-SAC motion controller drives the robot safely toward NBV while exploring the environment. Extensive experiments are conducted, with results indicating that our method has lower time and path costs than other methods.

The experiments also reveal several limitations of the current framework. First, the ACXCOOT traversal order is optimal only with respect to the frontiers known at the decision instant; because frontiers are revealed incrementally and the sensing range is limited, the global optimality of the overall exploration tour cannot be guaranteed, as observed in Env 10 of the simulation. Second, ETPP relies on sufficiently accurate localization: in the large real-world Env 4, severe localization drift prevented complete exploration, and a localization-quality monitoring mechanism is required for deployment in feature-sparse environments. Third, the method is designed for static environments, and dynamic obstacles with uncertain behavior remain challenging for the motion controller. Fourth, the roadmap quality depends on the node spacing $c_{V}$: an unsuitable setting makes the paths planned by IDijkstra more tortuous and increases the exploration cost, as analyzed in the simulation experiments. Finally, the APG-SAC policy is trained purely in simulation; although it transferred successfully in our real-world tests, systematic sim-to-real adaptation may be needed for substantially different robot platforms. These limitations define the directions of our future work: we will research traversal path planning for robots in dynamic unknown environments, paying particular attention to dynamic obstacles with uncertain behavior, and we will integrate localization-quality monitoring and sim-to-real adaptation strategies to strengthen the robustness of ETPP for large-scale deployments.

## Figures and Tables

**Figure 1:**
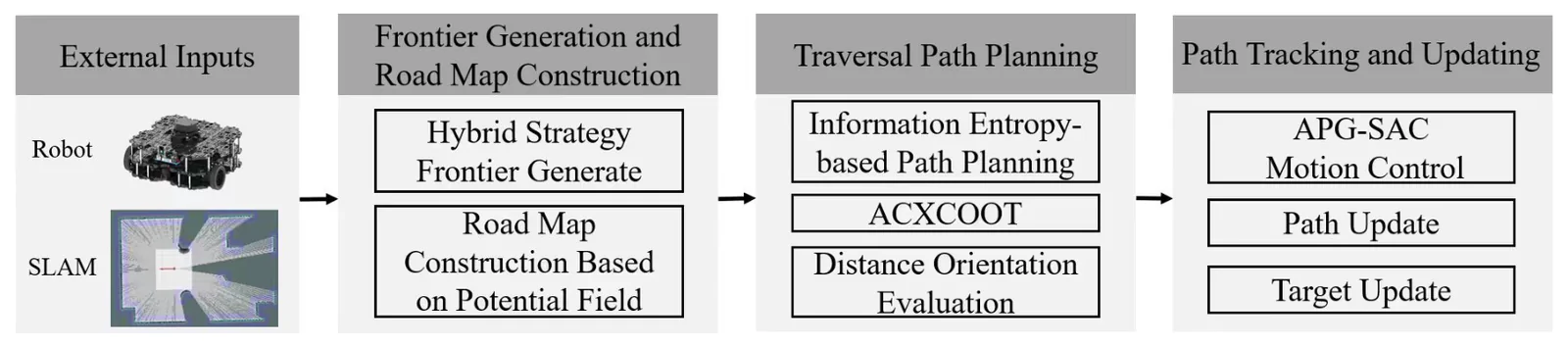
The framework of ETPP.

**Figure 2:**
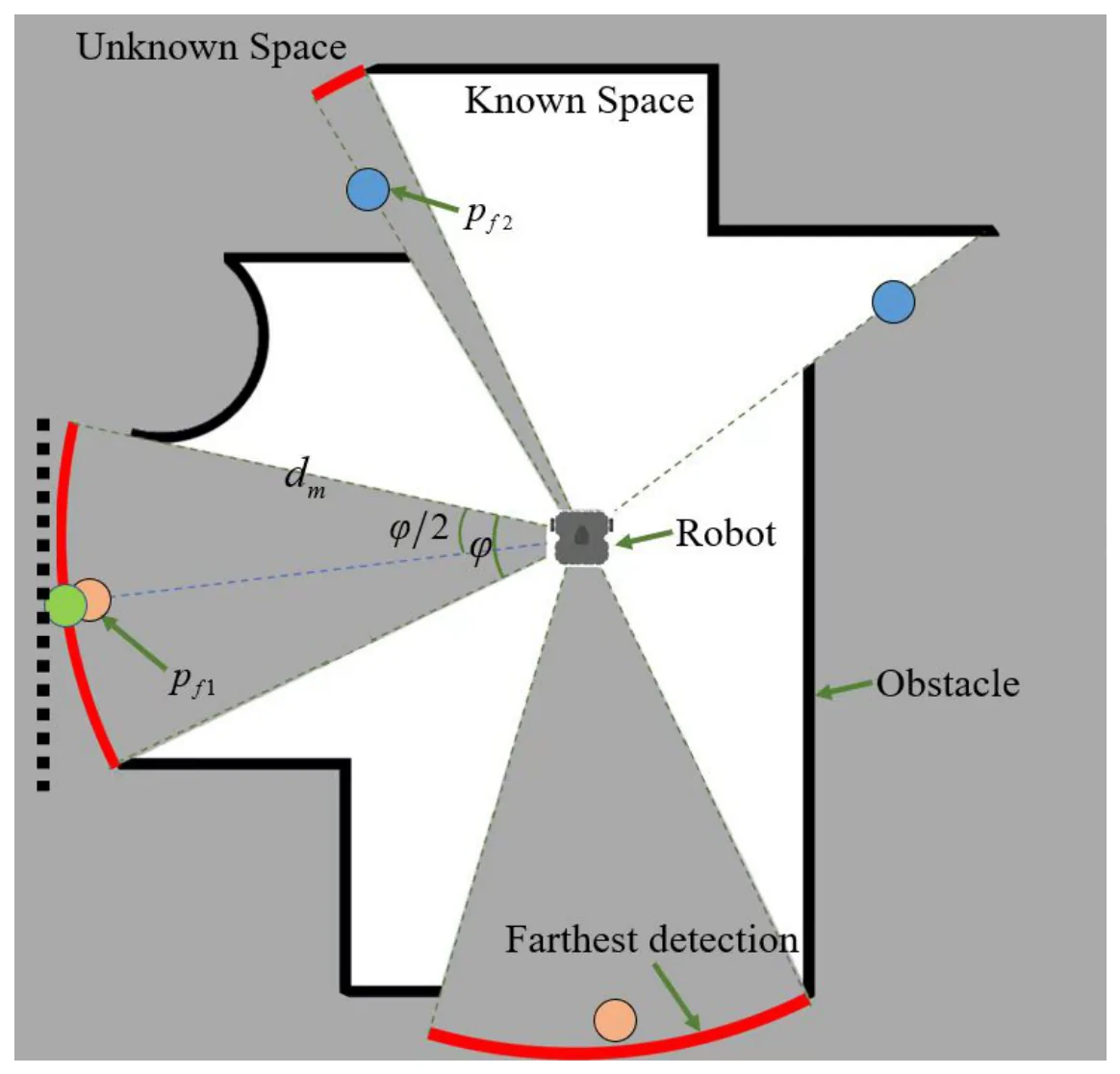
Frontier generation.

**Figure 3:**
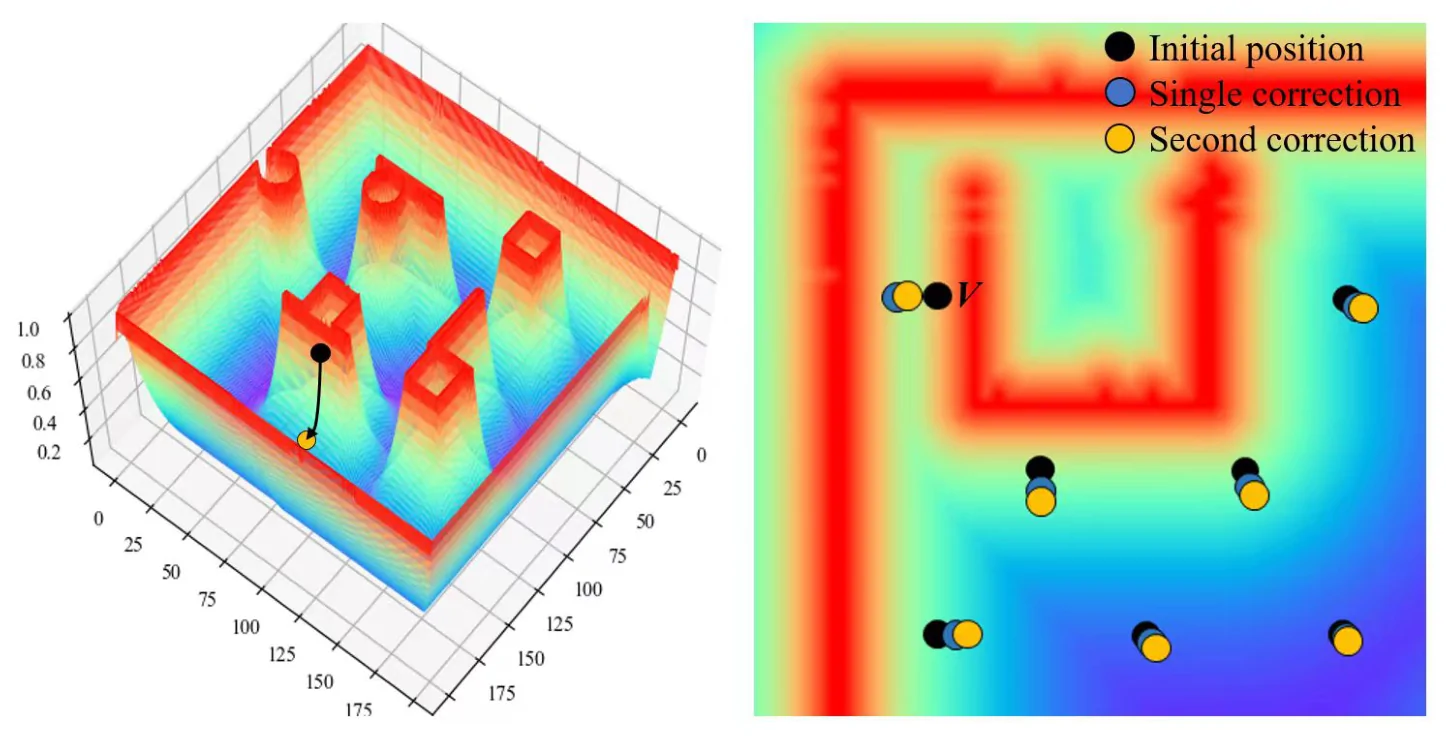
Road node position correction.

**Figure 4:**
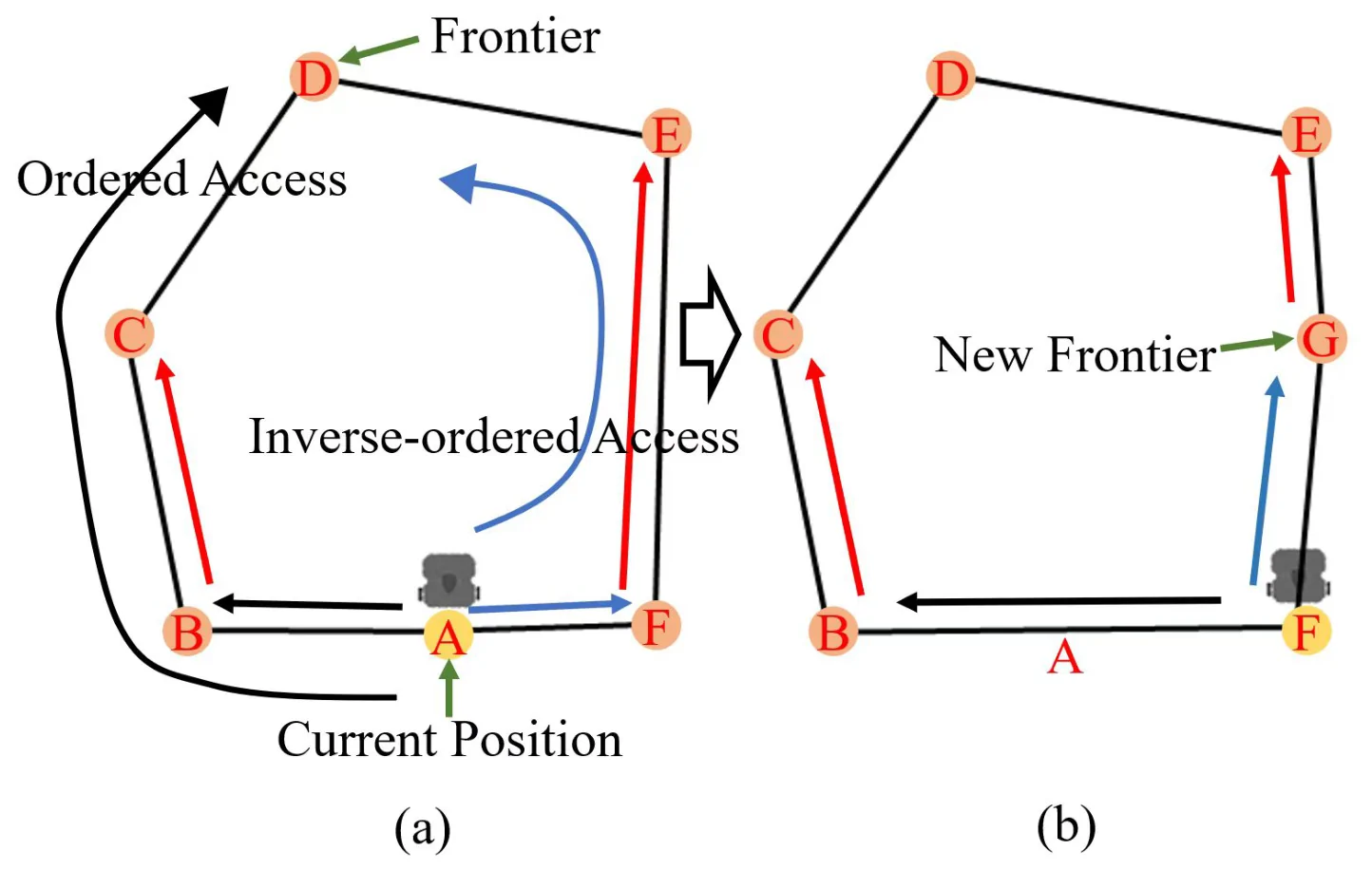
Traversal sequence of the frontiers: (**a**) the traversal sequence generated in the current exploration step; (**b**) the traversal sequence generated in the next exploration step.

**Figure 5:**
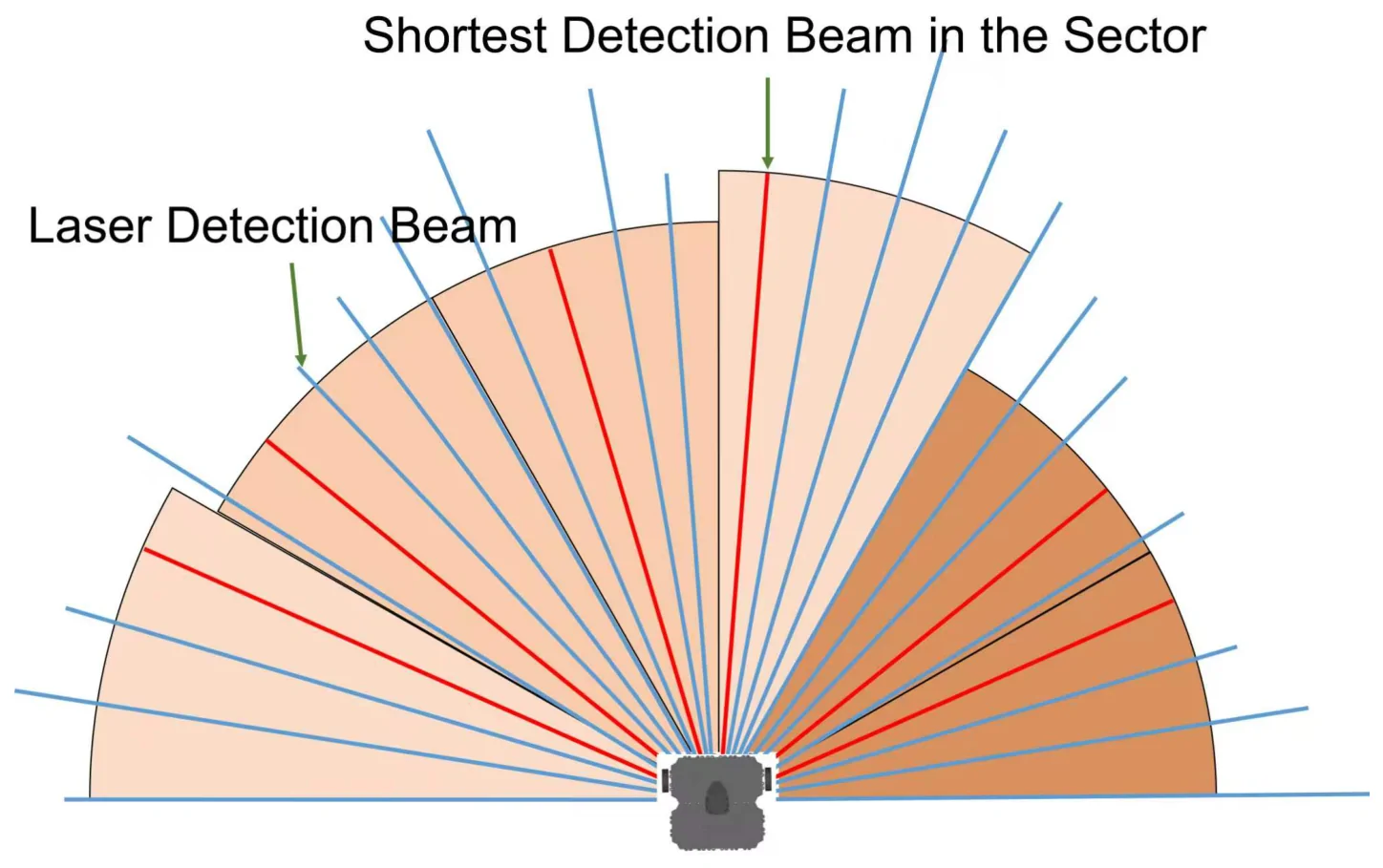
Angle pedestrian grid.

**Figure 6:**
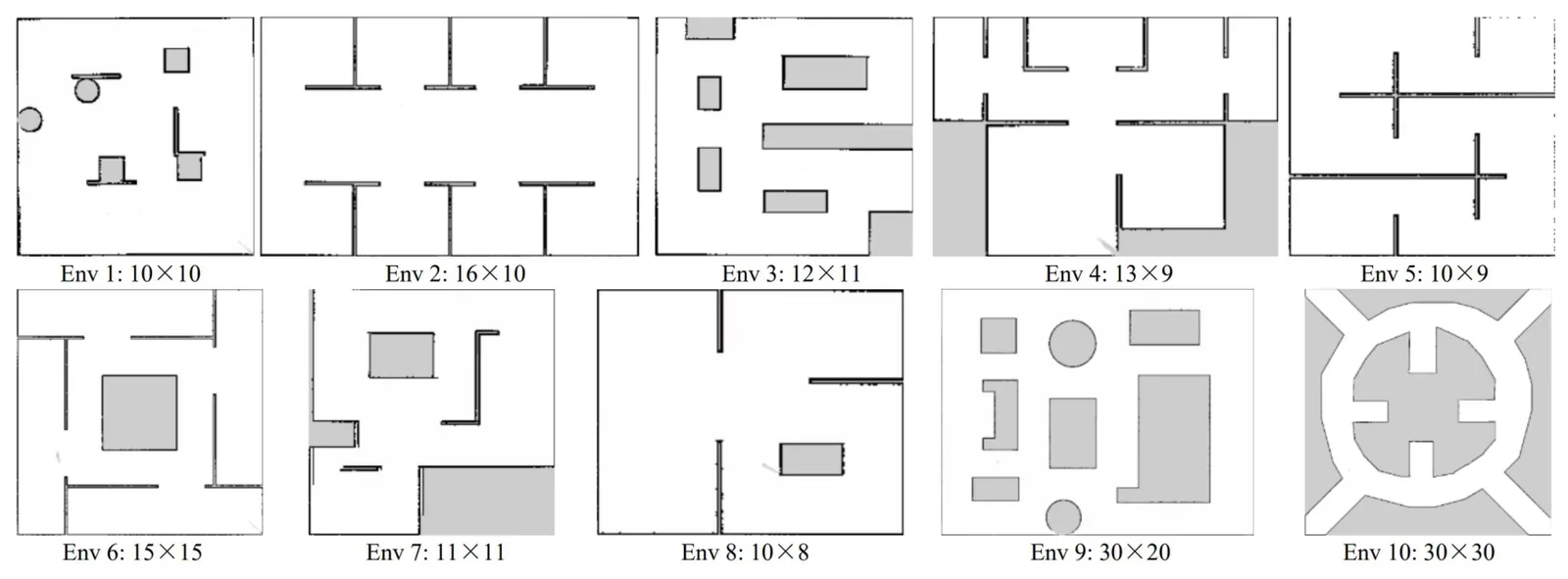
Exploration trajectories guided by ETPP (left) and Multi-RRT (right) methods in maze environment.

**Figure 7:**
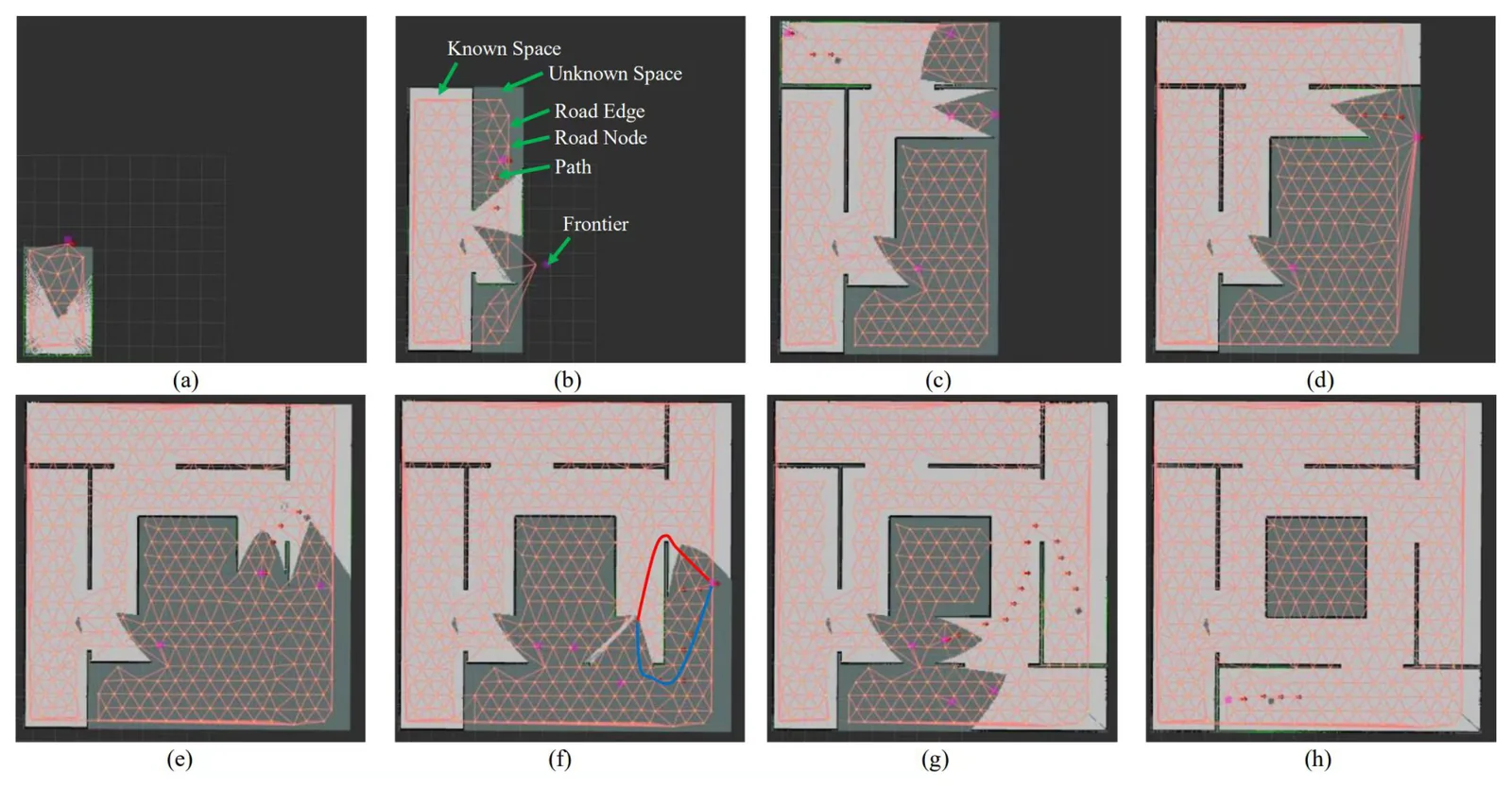
Autonomous exploration process using ETPP framework. (**a**) Initial exploration stage with generated frontiers; (**b**) road map construction; (**c**) global path planning on the road map; (**d**) the robot moving toward the NBV; (**e**) frontier regeneration during exploration; (**f**) planned path biased toward unknown areas; (**g**) continued exploration with updated frontiers; (**h**) real-time path update to bypass newly detected obstacles. Pink points: frontiers. Yellow points: road nodes. Pink edges: road edges. Red arrows: global path, the direction of the red arrow is meaningless.

**Figure 8:**
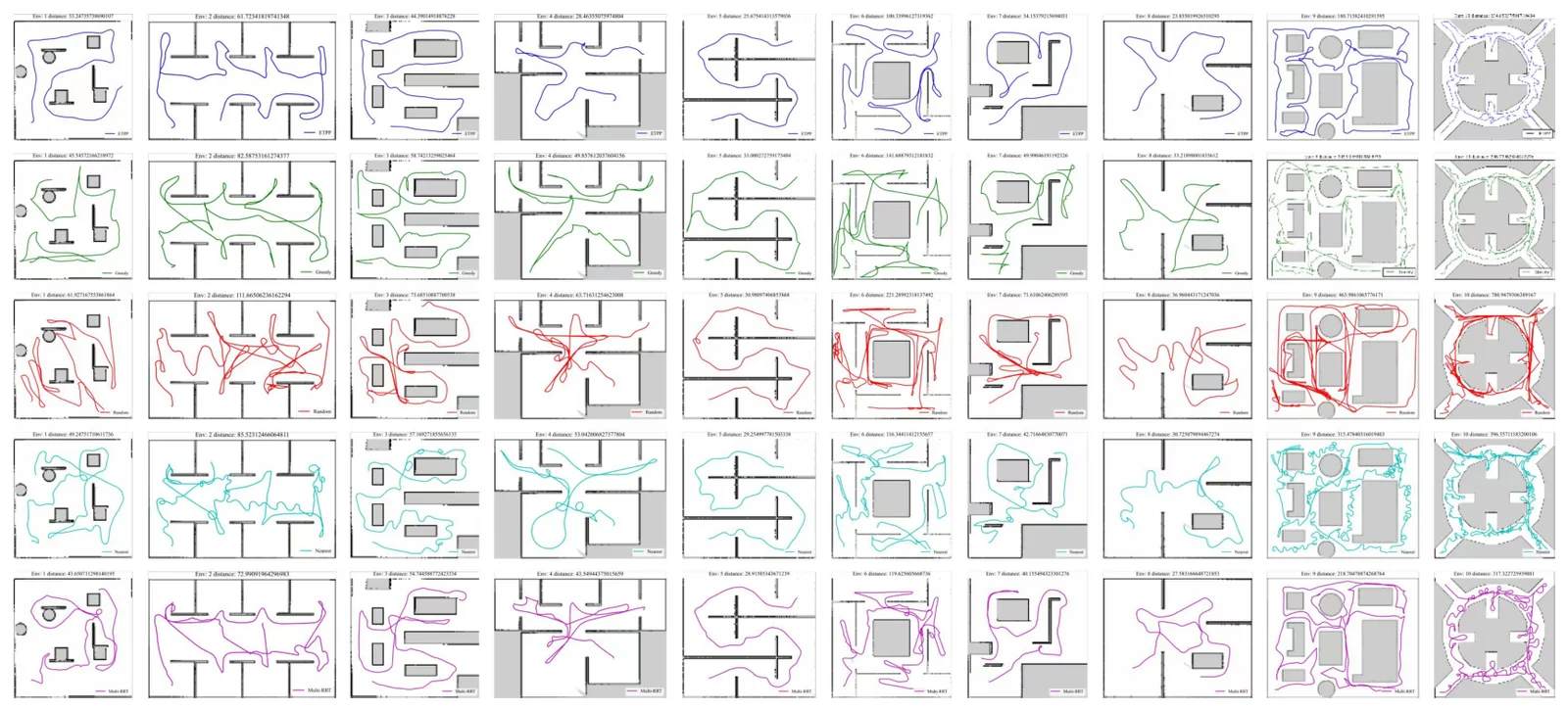
Explore trajectories of five methods in ten environments.

**Figure 9:**
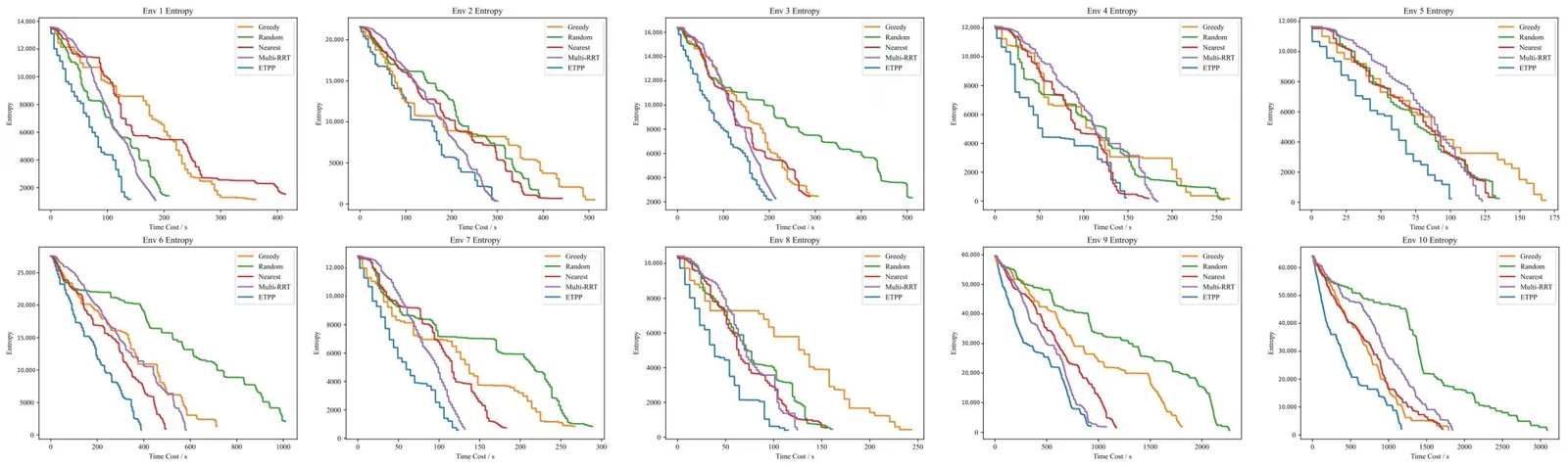
The exploration progress of five methods in ten environments.

**Figure 10:**
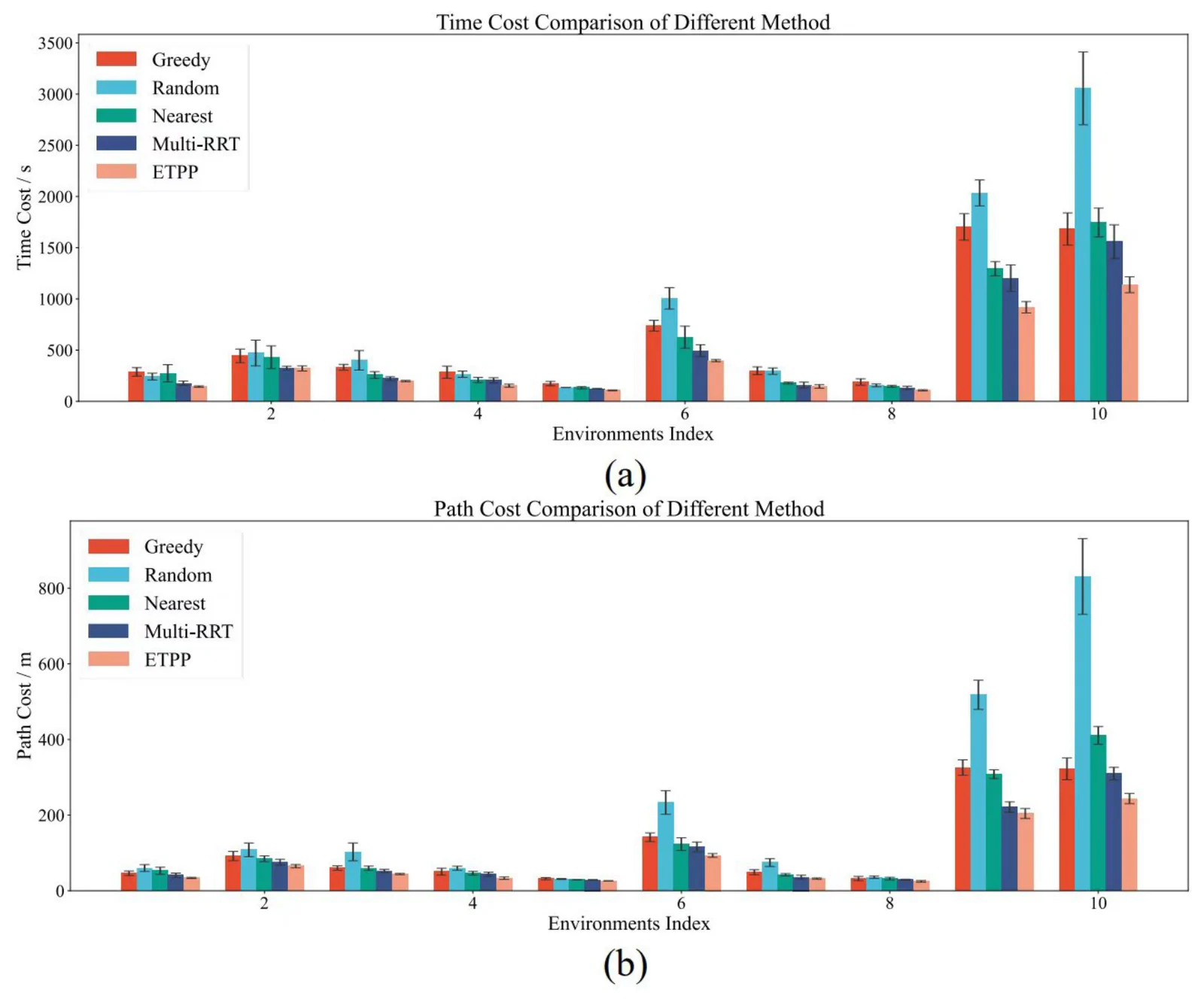
Time cost (**a**) and path cost (**b**) of five methods in ten environments.

**Figure 11:**
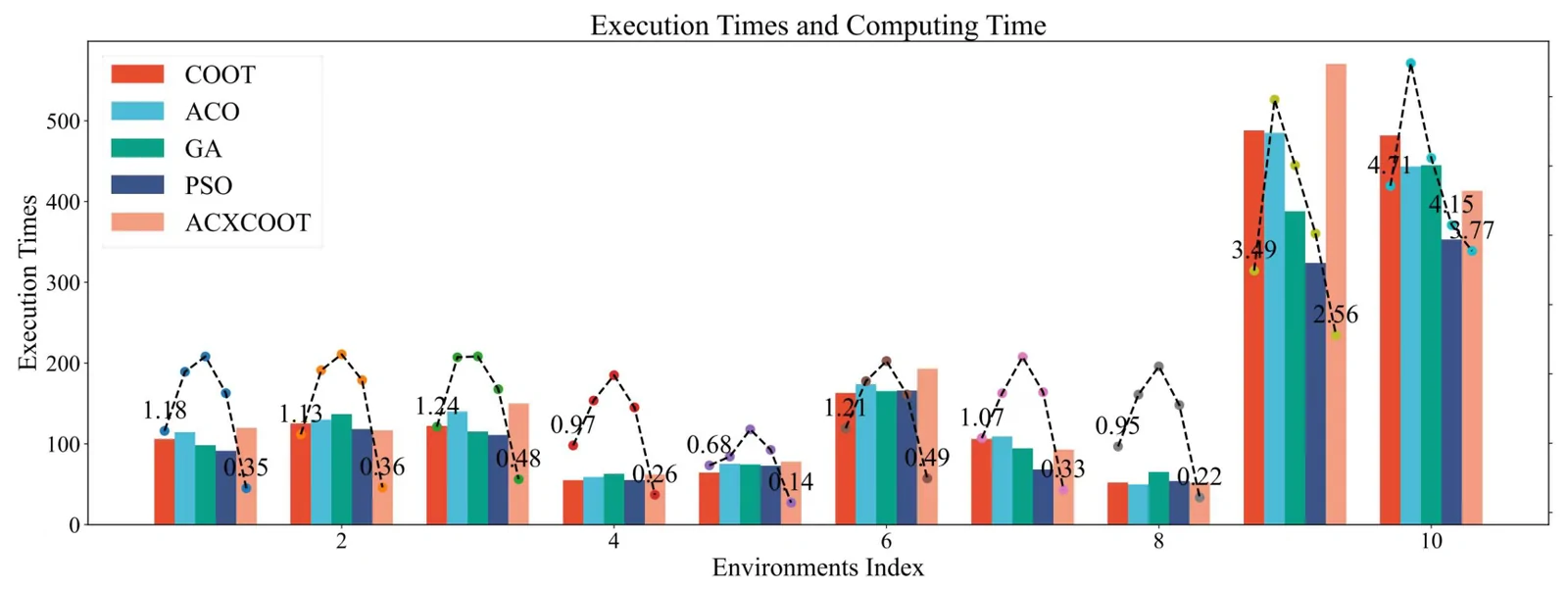
Comparison of the real-time performance of five intelligent optimization algorithms.

**Figure 12:**
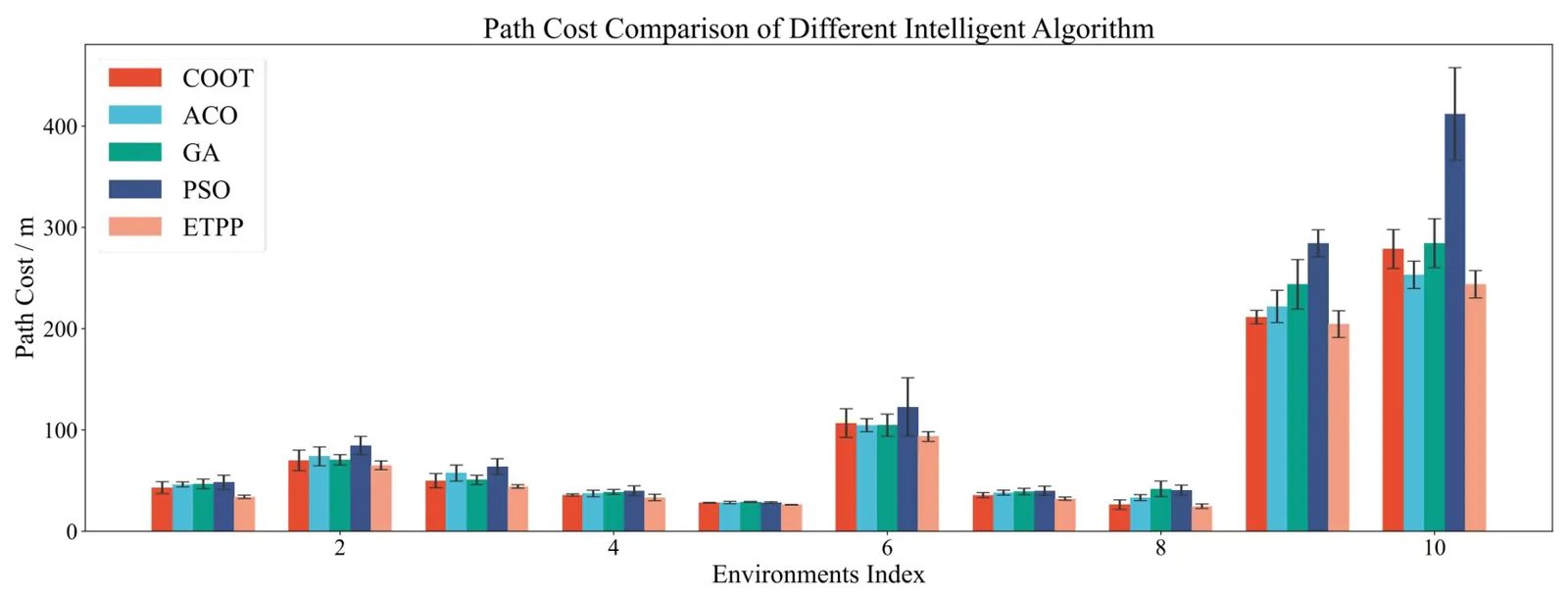
Comparison of path cost of five intelligent optimization algorithms.

**Figure 13:**
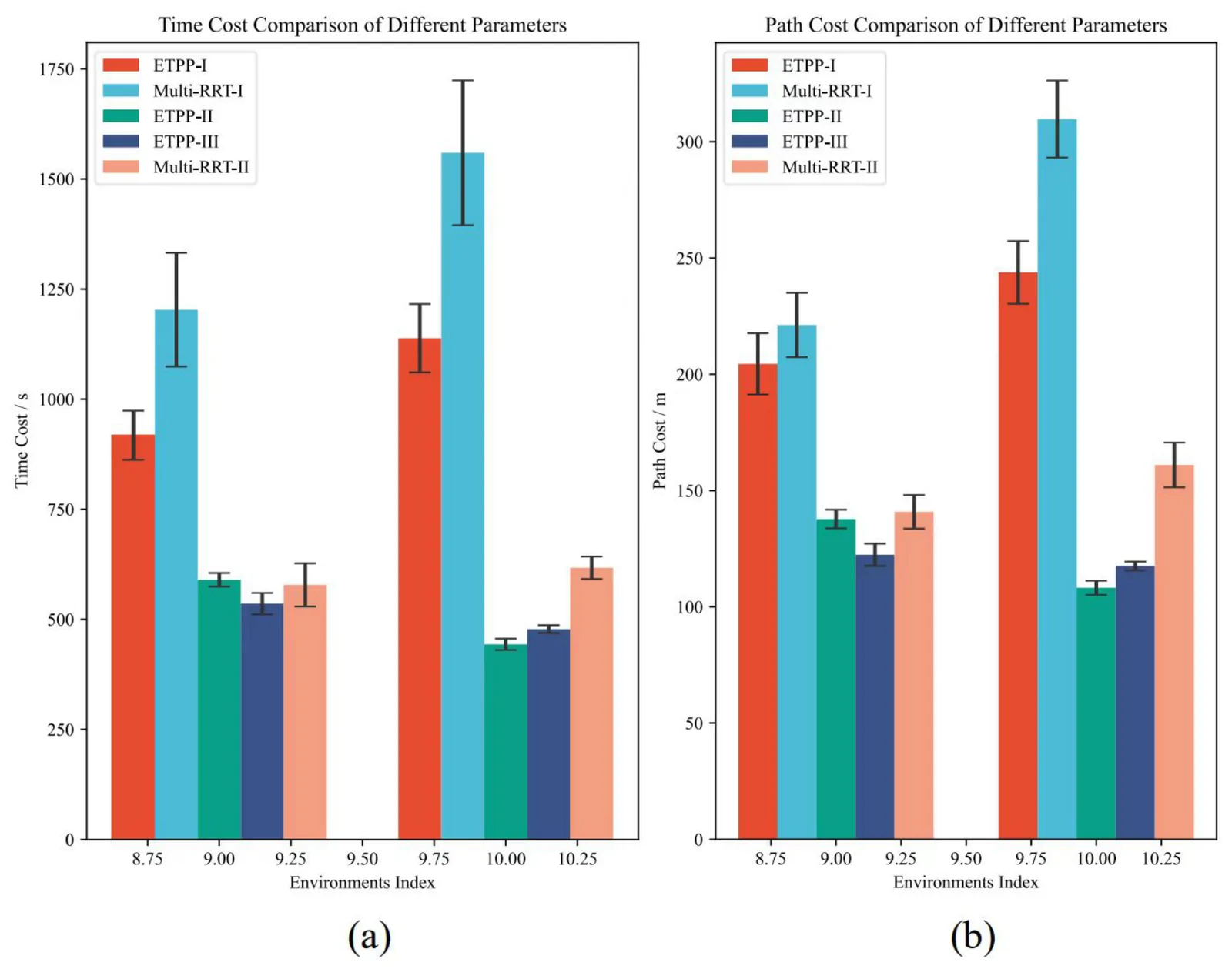
Exploration Costs of ETPP and Multi-RRT under Different Conditions in Env 9 and Env 10. (**a**) Time Cost. (**b**) Path Cost.

**Figure 14:**
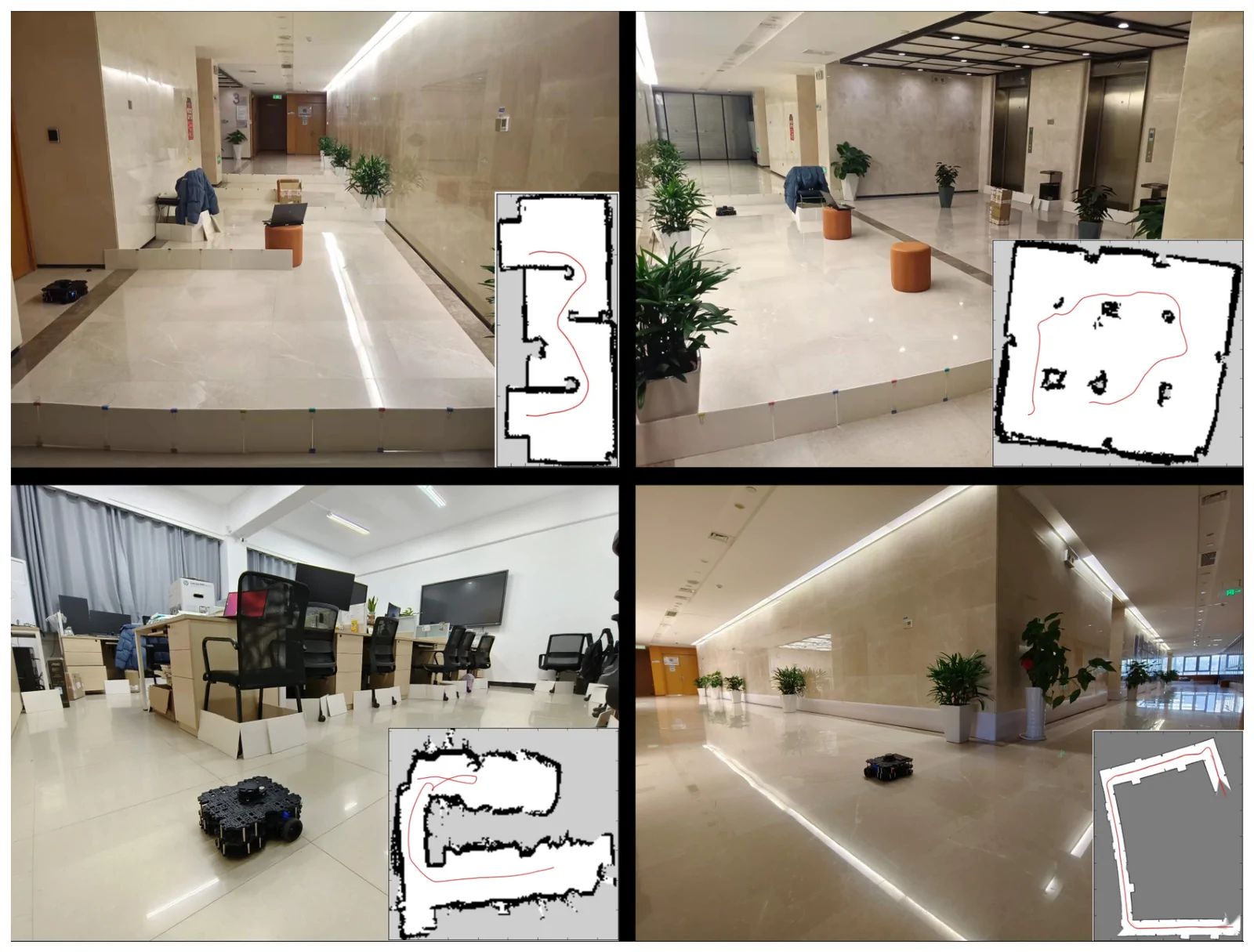
The exploration trajectories in four distinct real-world environments.

**Table 1:** Parameters set in experiments.

Parameter	Value	Parameter	Value	Parameter	Value
$n_{f1}$	25	$d_{m}$	3.5	$c_{1}$	0.2
$r_{\mathrm{bot}}$	0.21	$c_{V}$	0.8	$c_{s}$	10.0
${\mathrm{tol}}_{\mathrm{thr}}$	3	${\mathrm{pop}}_{\mathrm{size}}$	50	$l_{n}$	5
${\mathrm{Iter}}_{\max}$	500	$b_{1}$	0.45	$b_{2}$	0.93
$\chi_{1}$	0.4	$\chi_{2}$	0.6	$\varphi_{\min}$	0
$\varphi_{\max}$	359	$res_{s}$	1	$A_{s}$	50
$v_{\min}$	0	$v_{\max}$	0.26	$\omega_{\max}$	1.0
$I_{\mathrm{thr}}$	0.04				

**Table 2:** Experimental data on robot exploration in simulation environments: time cost (s).

Environment	Greedy	Random	Nearest	Multi-RRT	ETPP
Env 1 (10×10)	286.95	242.08	273.66	176.80	**143.96**
Env 2 (16×10)	444.37	472.06	430.70	323.07	**320.78**
Env 3 (12×11)	331.97	400.63	257.14	221.80	**198.22**
Env 4 (13×9)	284.59	264.54	208.77	204.47	**153.39**
Env 5 (10×9)	173.60	136.14	133.07	122.86	**107.35**
Env 6 (15×15)	738.55	1005.28	626.98	494.14	**397.79**
Env 7 (11×11)	298.70	294.67	178.50	159.18	**145.00**
Env 8 (10×8)	189.17	156.03	146.49	132.48	**107.26**
Env 9 (30×20)	1703.43	2034.75	1294.94	1203.14	**918.02**
Env 10 (30×30)	1682.45	3056.08	1745.57	1559.59	**1138.50**

Note: The best result in each environment is highlighted in bold.

**Table 3:** Experimental data on robot exploration in simulation environments: path cost (m).

Environment	Greedy	Random	Nearest	Multi-RRT	ETPP
Env 1 (10×10)	46.38	60.11	52.89	41.01	**33.95**
Env 2 (16×10)	97.76	108.25	84.72	75.57	**65.11**
Env 3 (12×11)	60.26	102.88	59.58	52.40	**44.25**
Env 4 (13×9)	50.57	59.64	46.27	43.67	**33.38**
Env 5 (10×9)	32.00	31.13	29.48	28.80	**26.18**
Env 6 (15×15)	141.49	233.37	123.37	115.83	**93.46**
Env 7 (11×11)	48.95	74.54	42.35	36.01	**32.19**
Env 8 (10×8)	32.50	35.76	32.18	29.32	**24.73**
Env 9 (30×20)	325.76	518.17	308.35	221.20	**204.48**
Env 10 (30×30)	322.56	830.99	410.78	309.78	**243.83**

Note: The best result in each environment is highlighted in bold.

**Table 4:** Algorithm parameters under different conditions.

Methods	$c_{V}$ (m)	$d_{m}$ (m)
Multi-RRT-I	—	3.5
Multi-RRT-II	—	7.0
ETPP-I	0.8	3.5
ETPP-II	0.8	7.0
ETPP-III	1.2	7.0

**Table 5:** Experimental data in real-world environments.

Environment	Map Size (m)	Time Cost (s)	Path Cost (m)	Speed (m/s)
Env 1	9×5	74.26	8.85	0.119
Env 2	8×8	121.75	14.61	0.120
Env 3	8×7	224.10	12.27	0.055
Env 4	34×23	715.81	73.94	0.103

## Data Availability

The data presented in this study are available on request from the corresponding author.

## Abbreviations

The following abbreviations are used in this manuscript:

ETPP	Efficient Traversal Path Planning
NBV	Next Best View
TSP	Traveling Salesman Problem
ACXCOOT	Adaptive Iterative Cyclic Crossover COOT
APG-SAC	Angle-based Pedestrian Grid Soft Actor–Critic
DRL	Deep Reinforcement Learning
RRT	Rapidly Exploring Random Tree
IDijkstra	Improved Dijkstra algorithm
OGM	Occupancy Grid Map
